# Enhancing Grasping Function with a Thermoresponsive Ionogel Adhesive Glove for Patients with Rheumatic Diseases

**DOI:** 10.1002/advs.202414761

**Published:** 2025-03-26

**Authors:** Shiqiang Wang, Shiqing Liu, Jingfeng Zhang, Zhouyang Yu, Yifan Shao, Cong Zhao, Jiahong Ma, Xin Fu, Mengqi Yang, Lie Chen, Mingjie Liu, Li Wen

**Affiliations:** ^1^ School of Mechanical Engineering and Automation Beihang University Beijing 100191 China; ^2^ Sino‐French Carbon Neutrality Research Center Ecole Centrale de Pekin/School of General Engineering Beihang University Beijing 100191 China; ^3^ Department of Rheumatology and Immunology Peking University Third Hospital Beijing 100191 China; ^4^ Key Laboratory of Bio‐Inspired Smart Interfacial Science and Technology of Ministry of Education School of Chemistry Beihang University Beijing 100191 China; ^5^ School of Automation Science and Electrical Engineering Beihang University Beijing 100191 China; ^6^ Department of Applied Chemistry College of Science China Agricultural University Beijing 100193 China

**Keywords:** hand exoskeleton, medical robotics, soft robotics, switchable adhesion, wearable robotics

## Abstract

Rheumatic diseases often result in joint deformities and peripheral nerve damage, leading to impaired hand function. Current wearable assistive gloves commonly rely on external forces to flex fingers for grasping, but they face limitations such as bulky hardware, complex finger manipulation, and a risk of joint injuries. This study presents a lightweight, portable, soft wearable adhesive glove based on thermoresponsive ionogels aimed at enhancing grasping function. The ionogel exhibits high adhesion (≈35 kPa to various materials) at 25 °C and low adhesion (≈6.8 kPa) at 45 °C. Smart adhesive pads use embedded flexible heaters and temperature sensors for closed‐loop control of the ionogels’ temperature, providing programmable adhesion. A rapid switch from high to low adhesion is achieved within 4 s at 4 V. Additionally, a hands‐free control interface uses inertial measurement units to detect the user's intent to release, facilitating easy and intuitive detachment. Weighing only 47 g, the glove is 7.2 times lighter than existing assistive gloves. Notably, it empowers users to grasp and release a variety of objects that will otherwise be unmanageable. Evaluation of various activities of daily living demonstrates that the glove significantly enhances grasping ability and increases autonomy for patients with rheumatic diseases.

## Graphical abstract

This study presents a novel portable grasp‐assist glove, weighing only 47 g. The glove integrates smart adhesive pads and a hands‐free control interface based on IMU, designed to aid rheumatic patients with impaired hand function. The system enables switchable adhesion with independent control and detection of user release intent. Preliminary clinical testing demonstrates its efficacy as a grasp‐assist device.

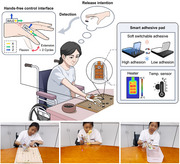

## 1 Introduction

Rheumatic diseases are a group of chronic autoimmune disorders primarily affecting the musculoskeletal system, including rheumatoid arthritis (RA), systemic lupus erythematosus (SLE), ankylosing spondylitis (AS), Sjögren's syndrome (SS), eosinophilic granulomatosis with polyangiitis (EGPA), among others.^[^ ^1^ ^]^ These diseases affect ≈5% of the global population, with both incidence and prevalence rates rising in recent years.^[^ ^2^ ^]^ Without timely treatment within two to three years, joint diseases like RA and AS can lead to joint deformities in up to 75% and 37% of patients, respectively, resulting in disability and workforce loss.^[^ ^1^, ^3^ ^]^ Peripheral neuropathy, a common complication, occurs in up to 50% of patients with RA, 18% with SLE, 25% with SS, and 65–80% with EGPA.^[^ ^4^ ^]^ This complication often manifests as motor or sensory impairments, significantly limiting patients' ability to perform activities of daily living (ADL).^[^ ^5^ ^]^ Given the frequent involvement of hand joints and peripheral nerves in rheumatic diseases, there is an urgent need to develop wearable assistive robotics to restore hand grasping functions in patients with severe hand impairments.

In recent decades, there has been a significant interest in developing hand‐assistive wearable robotics.^[^ ^6^, ^7^, ^8^ ^]^ These devices are primarily classified into two categories: rigid wearable hand exoskeletons and soft wearable assistive gloves. Rigid hand exoskeletons are bulky and heavy, consisting of motor‐driven rigid linkage mechanisms,^[^ ^9^, ^10^ ^]^ and often result in injury due to misalignment between the exoskeleton's rigid joints and the natural finger joints.^[^ ^11^, ^12^ ^]^ As a result, soft wearable assistive gloves have gained attention, offering a lighter weight and enhanced safety in human‐machine interaction.^[^ ^13^, ^14^, ^15^, ^16^ ^]^ Examples include hydraulic/pneumatic^[^ ^17^, ^18^, ^19^, ^20^, ^21^ ^]^ and motor‐cable^[^ ^22^, ^23^, ^24^, ^25^ ^]^ soft gloves, which use soft tubes and cables to bend fingers. Despite the glove's lighter weight, external hardware (such as pumps + tubes, motors + cables) is still required for actuation, making the overall system heavier (Table S1, Supporting Information). On the other hand, the working principle of these devices involves applying external force to bend the fingers around the object to achieve grasping. However, this approach has inherent limitations. For instance, forcibly bending the fingers can cause discomfort or pain, particularly for individuals with conditions such as arthritis. For patients with significant hypertonia or spasticity, the limited force generated by soft wearable assistive gloves may be insufficient to deform their “frozen” fingers.^[^ ^26^ ^]^ Increasing the driving force to compensate can easily cause finger injuries due to the relatively high joint compression force.^[^ ^8^, ^27^ ^]^ Moreover, although soft wearable assistive gloves have been widely explored to help patients with simple hand activities, the limited actuation degrees of freedom and lack of closed‐loop control with sensory feedback restrict the available grasping postures and the precision control of finger movements.^[^ ^8^, ^26^ ^]^ These limitations pose challenges in performing various fine grasping tasks in ADL. For example, 40% of ADL need pinch grasping,^[^ ^28^ ^]^ which involves handling thin and flat items, but most existing devices only enable cylindrical grasping.

A potential solution can be found in nature's toolbox of versatile grasping. Biological grippers rely on combinations of three basic grasping mechanisms: mechanical interlocking, friction, and adhesion.^[^ ^29^ ^]^ For example, human hands primarily rely on mechanical interlocking and friction rather than adhesion for grasping. In contrast, organisms like octopuses, tree frogs, and geckos utilize adhesion for grasping. Adhesion‐based grasping requires only minimal preload to generate significant adhesive force, eliminating the need for substantial closure force to grasp and manipulate objects.^[^ ^30^ ^]^ Additionally, because the adhesive force is normal to the object's surface, it enables additional handling strategies, such as single‐point grasping, which is impossible for human hands based on mechanical interlocking and friction. This characteristic simplifies the grasping process, obviating the need for fine grasping actions. For example, flat objects are often difficult to handle with finger‐like systems, but can be easily picked up by normal adhesion. Theoretically, these features could enable patients with hand disabilities to achieve a simplified grasping process and higher lifting force without the need for external actuating force on the fingers. Therefore, adhesion is a promising solution to address the limitations of soft wearable assistive gloves. Recently, several groups have begun to explore the integration of adhesion into soft wearable assistive gloves. Seokhwan Jeong et al. proposed a hand exoskeleton integrated with pneumatic self‐sealing suction cup modules to provide suction‐based grasping support.^[^ ^31^ ^]^ Similarly, Sean T. Frey et al. developed an octopus‐inspired wearable adhesive glove, which combines negative pressure adhesion and optical proximity sensors to detect objects and autonomously activate adhesion, facilitating the grasping and manipulation of underwater objects.^[^ ^32^ ^]^ However, due to reliance on pneumatic actuation, existing wearable adhesive gloves still cannot achieve compact untethered operation. Moreover, they are unable to detect the user's intent to release, thus failing to autonomously trigger detachment. Furthermore, clinical trials have not been conducted to validate the efficacy of wearable adhesive gloves in assisting patients with ADL.

**Figure 1:**
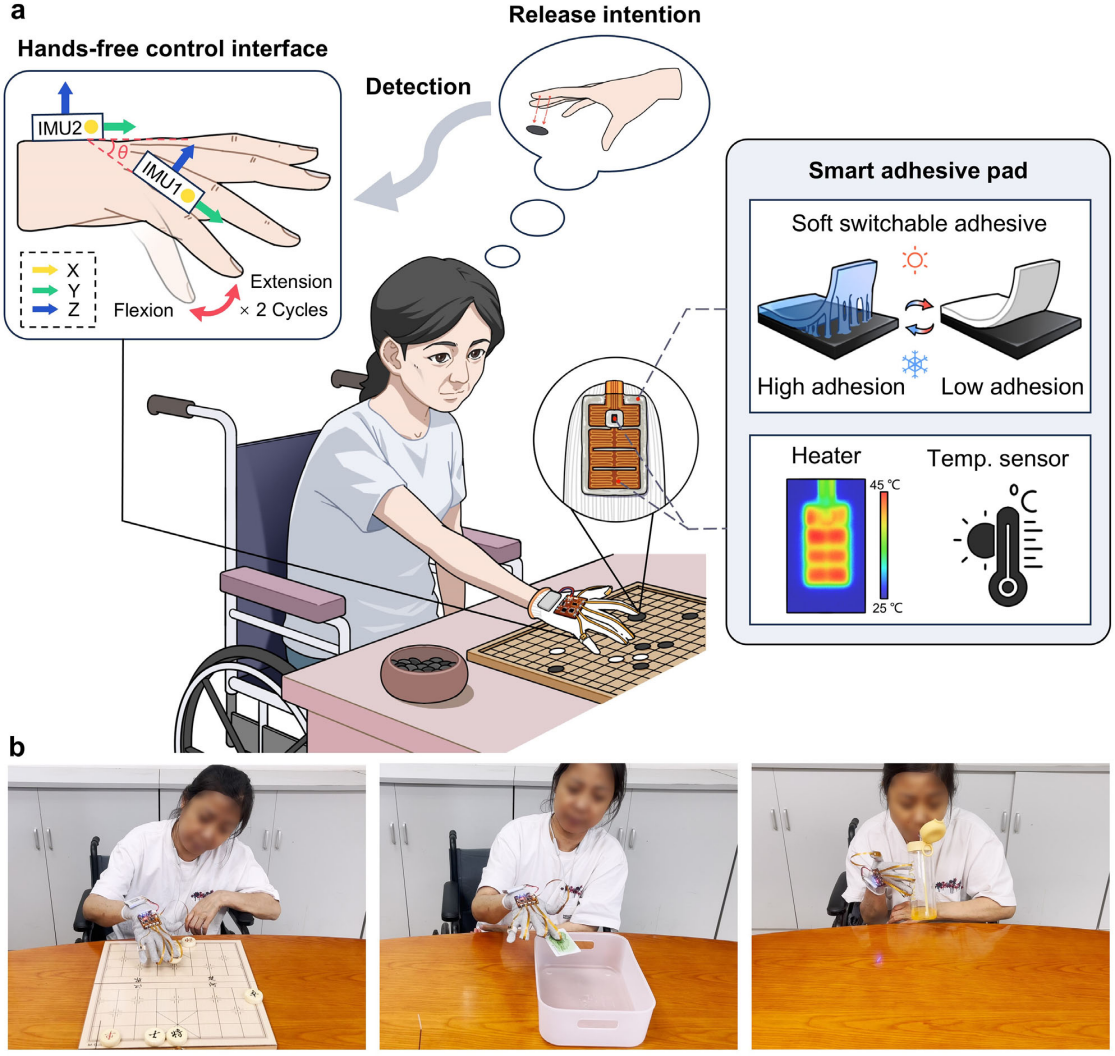
System overview. a) Schematic illustration showing the overall configuration and application of the portable grasp‐assist glove with soft switchable adhesives. b) Representative images of ADL tasks performed by a rheumatic patient with hand impairments using the glove, including playing Chinese chess, picking up a student card, and grasping a bottle for drinking.

**Figure 2:**
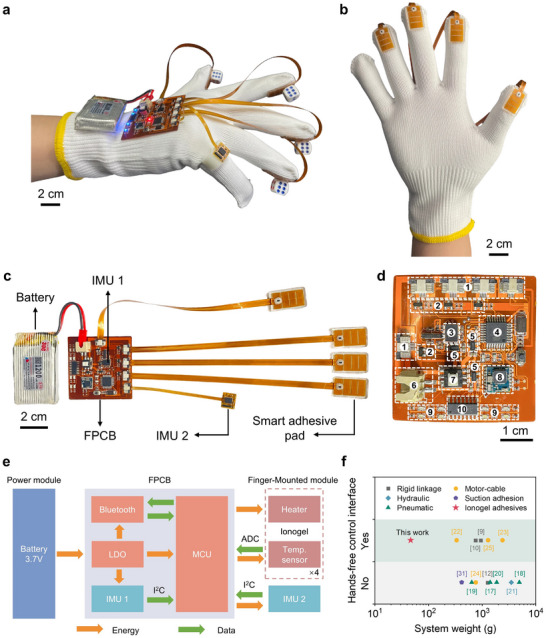
Overall design of the portable grasp‐assist glove with switchable soft adhesives. a) Side view of the proposed wearable adhesive glove. b) Palmar view of the proposed wearable adhesive glove. c) Optical image of a fully integrated electronic system. d) Photograph of the FPCB. The white dashed boxes indicate the locations of the major integrated circuit components. 1. FPC connectors. 2. NMOS transistor. 3. IMU. 4. MCU. 5. LDO. 6. To 3.7 V lithium polymer battery. 7. Switch. 8. Bluetooth. 9. LEDs. 10. Analog switch. e) Block diagram of the electronic system of the proposed robotic glove. f) Comparison of the control interface and system weight between various wearable assistive gloves. The proposed soft wearable adhesive glove has the hands‐free control interface and the lightest system weight (47 g).

To overcome the limitations of existing research, we present a novel portable grasp‐assist glove. This glove integrates smart adhesive pads and a hands‐free control interface based on inertial measurement unit (IMU) to aid rheumatic patients with impaired hand function, as illustrated in **Figure** **1**. The system is an iteration of a previous version,^[^ ^33^ ^]^ featuring switchable adhesion with independent control and detection of user release intent. The smart adhesive pads, distributed at the fingertips, consist of thermoresponsive ionogel with switchable adhesion and a flexible heater integrated with an in situ temperature sensor to assist in grasping. At a voltage of 4 V, the adhesion switching from high to low occurs in ≈4 s. Two consecutive flexion and extension movements of the little finger are interpreted as the user's intention to release an object, which can be detected in real‐time by the hands‐free control interface, triggering heating for detachment. This interface allows for easy and intuitive detachment control without needing assistance from the other hand. The glove is highly portable, with a total system weight of only 47 g (Figure S1 and Table S2, Supporting Information). To our knowledge, this glove is the lightest among reported wearable assistive gloves concerning total system weight (Figure 2f; and Table S1, Supporting Information). An experimental evaluation with a rheumatic patient demonstrated that the glove successfully enabled the completion of various simulated ADL grasping tasks, establishing its efficacy as an assistive grasping device.

## 2 Results

### 2.1 System Overview

The proposed wearable assistive glove with soft switchable adhesives for assisting rheumatic patients in ADL is demonstrated in **Figure** **2**. As shown in Figure 2a, the glove comprises five key components: four smart adhesive pads, a hands‐free control interface based on IMU, a flexible printed circuit board (FPCB), a rechargeable lithium‐polymer battery, and a commercially available textile glove. Specifically, the smart adhesive pad consists of a thermoresponsive ionogel with switchable adhesion, embedded with a flexible heater and in situ temperature sensor. At room temperature, the pad exhibits high adhesion, enabling it to grasp objects effectively. Upon heating, the adhesive force decreases, facilitating the release of objects. As shown in Figure 2b, the smart adhesive pads are adhered to the four fingers (thumb, index, middle, and ring fingers) of the glove using silicone adhesive, allowing independent control of the switchable adhesion for each finger. The IMU‐based hands‐free control interface uses two consecutive flexion and extension movements of the little finger as a trigger signal for object release, providing a simple and intuitive control mechanism. The finger‐mounted IMU module is attached to the little finger using a 3D‐printed flexible adapter, which works together with the IMU on the FPCB to capture the little finger's movements, thereby detecting the user's intent to release an object. The lithium‐polymer battery (52 × 30 × 9 mm) and the FPCB (48 × 45 × 0.07 mm) are adhered to the back of the glove with double‐sided tape, providing power and control for the glove. The ultrathin characteristic of the FPCB offers significant flexibility (Figure S2, Supporting Information), allowing it to conform to the user's hand shape for enhanced comfort during wear. The glove features a modular design with compact size, lightweight, and high maintainability, ensuring portability and ease of use. With the help of another person, the glove can be donned or doffed by the patient in ≈2 min.

Figure 2c,e illustrates the optical image and block diagram of the electronic system, respectively. The custom‐designed FPCB incorporates a microcontroller unit (MCU), an IMU sensor module, a Bluetooth low‐energy (BLE) wireless transmission module, and LEDs, among other components. Additionally, the FPCB provides physical interfaces for four smart adhesive pads, an finger‐mounted IMU module, and a lithium‐polymer battery, enabling a modular design. The detailed optical image and circuit schematic diagram of the FPCB can be found in Figure 2d and Figure S3 (Supporting Information). The lithium‐polymer battery (3.7 V, 1200 mAh) supplies a stable voltage (3.3 V) to the MCU, BLE module, and IMU module via low dropout regulators (LDOs). The MCU regulates the heating power of the flexible heater by applying the pulse width modulation (PWM) signal to the N‐type metal‐oxide‐semiconductor (NMOS) transistor, while employing real‐time temperature feedback from the temperature sensor for closed‐loop control of the ionogel's temperature. Two nine‐axis degrees of freedom IMUs accurately monitor the joint angles of the little finger to detect the user's intent to release an object. Temperature sensor data are digitized via a 10‐bit analog‐to‐digital converter (ADC) and sent to the MCU, while IMU sensor data are sent through the inter‐integrated circuit (I^2^C) bus to the MCU for processing. The LEDs on the FPCB provide visual feedback on the system status to the user. The BLE module wirelessly transmits temperature and IMU sensor data in real time to a laptop for further analysis.

### 2.2 Design, Mechanism, and Characterization of Thermoresponsive Ionogels with Switchable Adhesion

**Figure 3:**
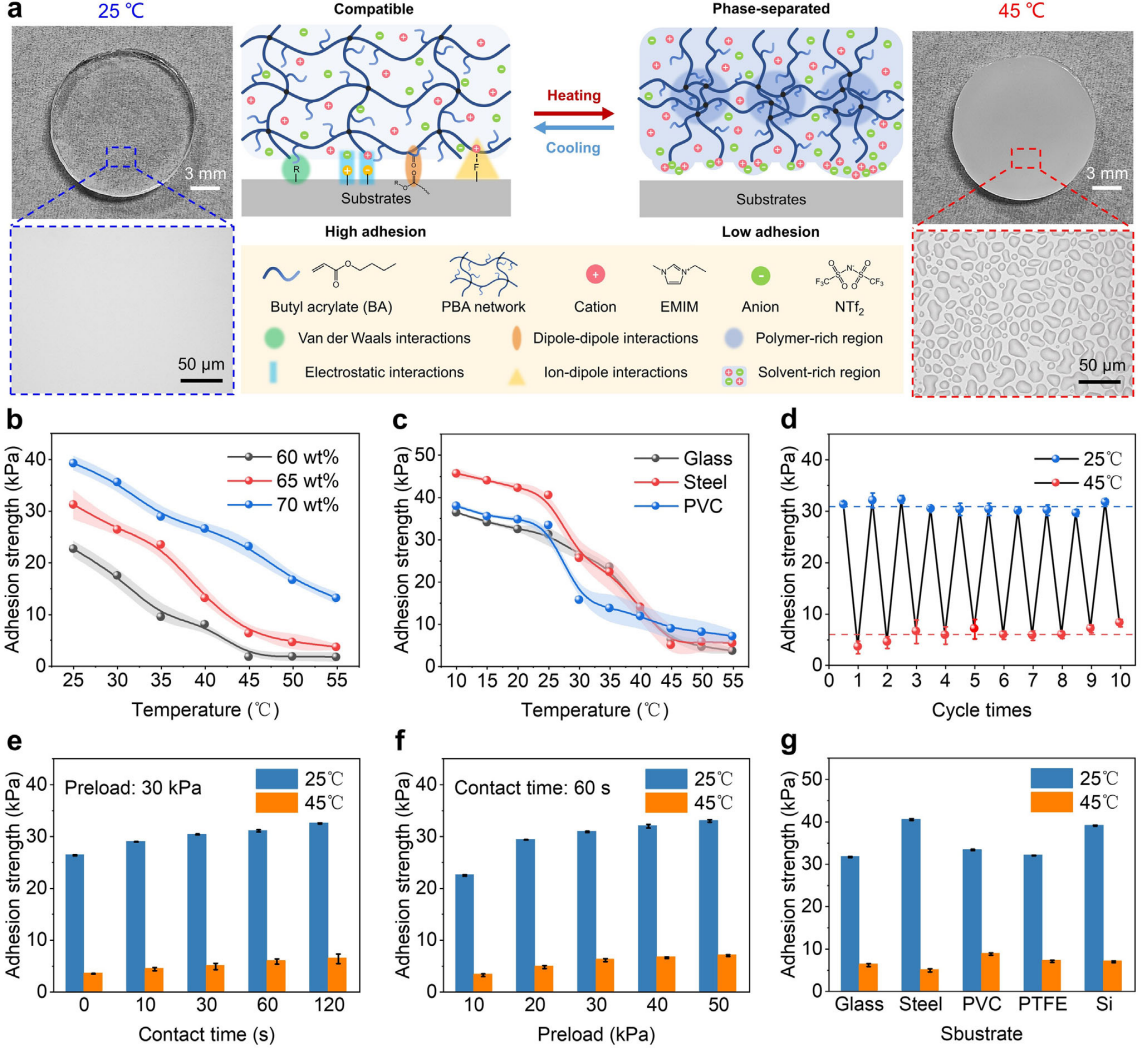
Design, mechanism, and characterization of thermoresponsive ionogels with switchable adhesion. a) The proposed mechanism of thermoresponsive ionogels with switchable adhesion. Left: photo of ionogel disc at 25 °C and optical microscopic image of the ionogel surface. Middle: schematic illustration showing thermoresponsive adhesion switching mechanism of ionogels. Right: photo of ionogel disc at 45 °C and optical microscopic image of the ionogel surface. b) Temperature‐dependent adhesion strengths of the ionogels with 60, 65, and 70 wt.% PBA contents against glass substrates. c) Adhesion strengths of the ionogel with 65 wt.% PBA content against various substrates at different temperatures. d) Cycling adhesion tests of the ionogels between 25 and 45 °C against glass substrates. e) The effects of contact time on adhesion strength for the ionogels at high and low temperatures against glass substrates. f) The effects of preload on adhesion strength for the ionogels at high and low temperatures against glass substrates. g) Thermoresponsive adhesion switching for the ionogels is available on various substrates.

The ionogel is synthesized via a one‐step photopolymerization of a precursor solution containing monomer (butyl acrylate, BA), cross‐linker (ethyleneglycol dimethacrylate, EGDMA), photoinitiator (diethoxyacetophenone, DEOP), and ionic liquid (1‐ethyl‐3‐methylimidazolium bis(trifluoromethylsulfonyl)imide, [EMIM][NTf_2_]). Details on preparing the ionogel precursor solution are provided in the Experimental Section. The thermoresponsive switchable adhesion of the ionogel is illustrated in **Figure** **3**a. The ionogel exhibits high adhesion to the substrate below the lower critical solution temperature (LCST) and low adhesion to the substrate above the LCST. This switchable adhesion is induced by LCST phase separation.^[^ ^33^, ^34^ ^]^ Below the LCST, the butyl side chains on the ionogel surface interact extensively with the substrate through multiple mechanisms, including dipole‐dipole interactions, ion‐dipole interactions, electrostatic interactions, and van der Waals interactions, thereby imparting high adhesion.^[^ ^35^ ^]^ Above the LCST, phase separation occurs, leading to low adhesion. This is due to two factors: first, the phase separation causes the butyl side chains on the ionogel surface to retract, weakening their interaction with the substrate. Second, the extruded ionic liquid microdroplets on ionogel surfaces due to phase separation inhibit contact between the butyl side chains and the substrate. This thermoresponsive phase behavior of the ionogel is reversible; upon cooling, the extruded microdroplets are reabsorbed, restoring the ionogel's transparency and high adhesion.

To develop ionogels suitable for wearable adhesive gloves, we investigated the effect of polymer content on adhesive performance. Pull‐off tests were conducted to measure the adhesion strength of ionogels containing 60 to 70 wt.% poly(butyl acrylate) (PBA) on glass substrates at temperatures ranging from 25 to 55 °C (Figure 3b). Unless otherwise specified, all pull‐off test conditions were 60 s contact time and 30 kPa preload. The experimental setup for the pull‐off test is shown in Figure S4 (Supporting Information). The adhesion strength of the ionogels increased at both low and high temperatures with higher PBA content. Specifically, as the PBA content increased from 60 to 70 wt.%, the adhesion strength at 25 °C (room temperature) increased from 22.6 to 39.3, and at 45 °C (the threshold for low‐temperature burns) it increased from 1.6 to 23.1 kPa. This enhancement in adhesion can be attributed to two factors. First, the increased polymer content enhances the internal cohesion of the ionogel and improves interfacial adhesion by providing more butyl side chains on the ionogel surface. Second, the reduction in ionic liquid content diminishes its adverse effect on adhesion strength during phase separation. Moreover, increasing the PBA content from 60 to 70 wt.% raised the LCST from 32 to 40 °C (Figure S5, Supporting Information). This indicates that ionogels with higher PBA content require a higher temperature for adhesion switching, which increases both the switching time and the risk of burns. Therefore, ionogels suitable for wearable adhesive gloves should exhibit high adhesion strength at room temperature, low adhesion strength at 45 °C, and an appropriate LCST. Considering these requirements, we selected the ionogel with 65 wt.% PBA, which has an LCST of 38 °C, and exhibits adhesion strengths of 31.2 kPa at 25 °C and 6.3 kPa at 45 °C on glass substrates.

As shown in Figure 3c, we evaluated the temperature‐dependent adhesion strength of the ionogel containing 65 wt.% PBA on various substrates, including glass, stainless steel, and polyvinyl chloride (PVC). At temperatures ranging from 10 to 25 °C, the ionogel exhibited high adhesion strengths on all substrates (>31 kPa), indicating its ability to maintain strong adhesion in low‐temperature environments. As the temperature increased, the adhesion strength rapidly decreased, stabilizing at a lower value at 45 °C. To assess the repeatability of the ionogel's reversible adhesion, cycling experiments were performed (Figure 3d). After 10 heating‐cooling cycles on a glass substrate, the ionogel maintained stable performance within the measurement error, demonstrating its switchability and repeatability. When the number of adhesion cycles was increased to 100, the adhesion strength of the ionogels remained stable and reversible, with the extrusion of ionic liquid microdroplets induced by phase separation still observable (Figure S6, Supporting Information). The effects of contact time and preload on the adhesion strength of the ionogel were also investigated (Figure 3e,f). At both 25 and 45 °C, the adhesion strength increased with longer contact times (constant preload: 30 kPa) and higher preload (constant contact time: 60 s). However, beyond 60 s of contact time and 30 kPa of preload, further increases in these parameters resulted in negligible improvements in adhesion strength. This can be attributed to the enhanced contact between the ionogel and the substrate under longer contact times and higher preload, which maximizes adhesion strength.^[^ ^36^, ^37^ ^]^ Additionally, the thermoresponsive switchable adhesion of the ionogel was effective across various substrates, including glass, steel, PVC, poly(tetrafluoroethylene) (PTFE), and Si (Figure 3g). At 25 °C, the ionogel exhibited high adhesion strengths of ≈35 kPa on these materials, while at 45 °C, the adhesion strength decreased to ≈6.8 kPa. Furthermore, the impact of environmental factors on the adhesion strength of ionogels has been investigated, including surface wetting and dust pollution. Compared to dry surface conditions, the adhesion strength of ionogels slightly decreased at 25 °C when wetted, while remaining nearly unchanged at 45 °C (Figure S7, Supporting Information). This indicates a degree of robustness under wet conditions. The minimal impact of surface wetting on the adhesion strength of ionogels can be attributed to the hydrophobic components (PBA network and ionic liquids) in ionogels.^[^ ^34^ ^]^ As shown in Figure S8 (Supporting Information), the adhesion strength of ionogels slightly decreased when the surface was polluted by a small amount of dust. In extreme cases of heavy dust pollution, the adhesion strength experienced a significant reduction. However, after cleaning with fresh ionogels, a substantial recovery in adhesion strength could be achieved.

### 2.3 Design and Characterization of Smart Adhesive Pads

The smart adhesive pad is composed of an ionogel embedded with a flexible heater integrated with an in situ temperature sensor. The flexible heater, featuring an all‐in‐one design, enables closed‐loop heating and in situ temperature monitoring. The heater adopts a simple trilayer configuration consisting of polyimide (PI) (12.5 µm thick), copper (Cu) (18 µm thick), and an additional PI layer (12.5 µm thick). The PI layers electrically insulate the Cu layer and position it at the neutral mechanical plane. The heating element of the flexible heater is formed by a serpentine Cu trace (width: 0.25 mm), distributed over an area of 22 × 14.4 mm^2^. Portions of the heating element are hollowed out to facilitate enhanced bonding between the upper and lower layers of the ionogel. The total electrical resistance of the flexible heater is ≈1.2 Ω, with detailed geometrical specifications provided in Figure S9 (Supporting Information). The temperature sensor (MCP9700, Microchip Technology, USA) enables real‐time and in situ monitoring of the ionogel's temperature, providing feedback for precise temperature control. To enhance the sensor's responsiveness during rapid temperature changes and to offer insulation for the sensor pins, thermally conductive (TC) silicone (K‐5207, Kafuter, China) with a thermal conductivity of 2.5 W m^−1^ K^−1^ is applied around the sensor.

**Figure 4:**
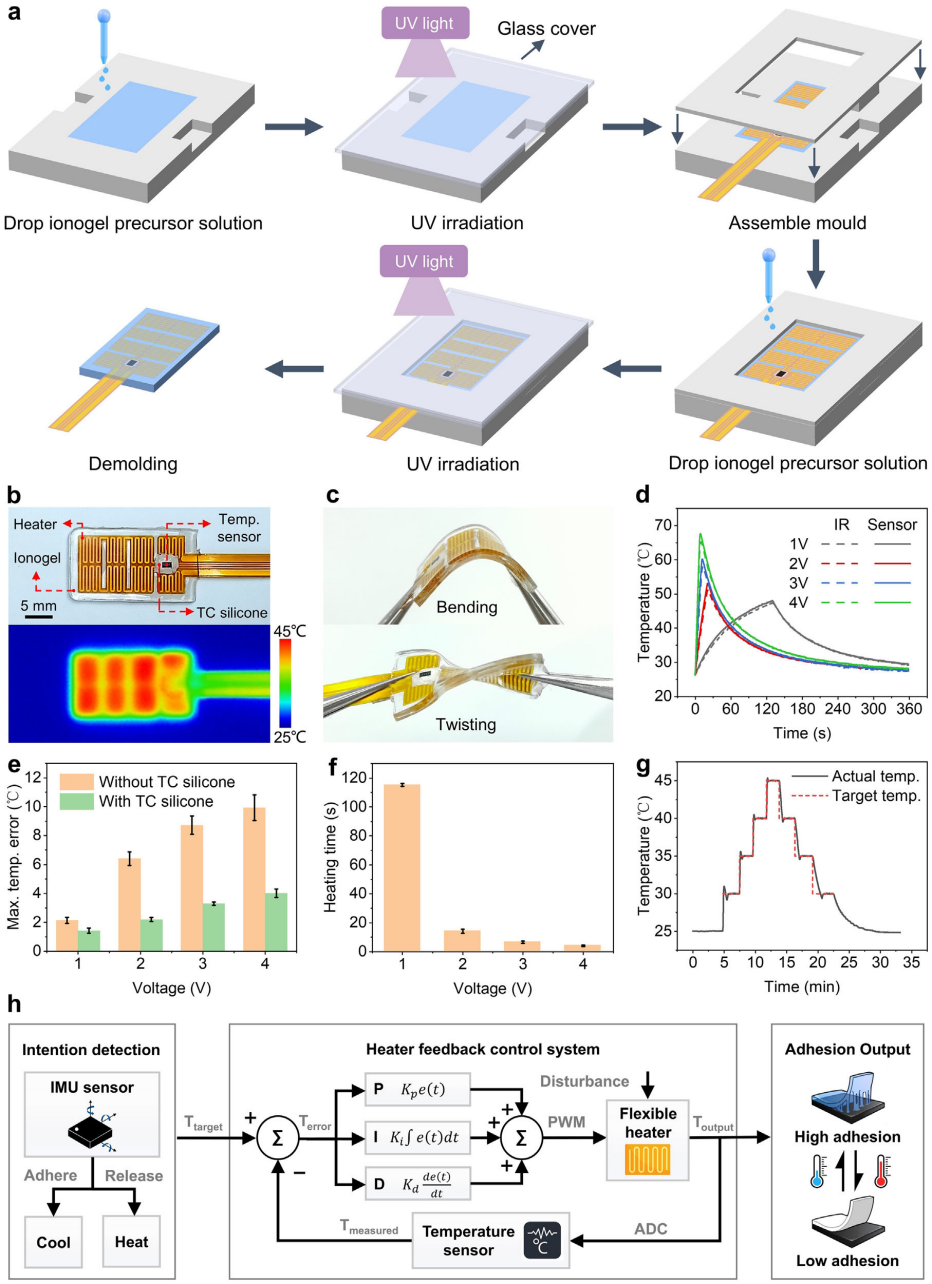
Smart adhesive pads with the flexible heater and in situ temperature sensor embedded. a) Schematic illustration of fabrication steps for smart adhesive pads. b) Optical image and IR camera image of the smart adhesive pad. c) Photographs of the smart adhesive pad under bending and twisting. d) The temperature evolution of the smart adhesive pad measured via the in situ temperature sensor and the IR camera at different applied voltages (1, 2, 3, and 4 V). Recorded temperature traces of the in situ temperature sensor (solid lines) are in excellent agreement with data taken from IR images (dashed lines). e) Maximum temperature error between the in situ temperature sensor with and without thermally conductive silicone applied and the infrared camera during heating from room temperature to 45 °C under various voltages. f) Heating time required for the smart adhesive pad to heat from room temperature (25 °C) to 45 °C by the flexible heater at different applied voltages. g) Time‐dependent temperature profile of the smart adhesive pad programmed to modulate the temperature in a stepwise fashion realized by the PID controller at ambient temperature (25 °C). h) PID control scheme for closed‐loop temperature control.

**Figure** **4**a outlines the key steps for embedding the flexible heater, integrated with an in situ temperature sensor, into the ionogel to fabricate the smart adhesive pad. First, the ionogel precursor solution is poured into a CNC‐machined PTFE lower mold, covered with a glass slide, and exposed to UV light (365 nm, 800 mW cm^−2^) for 10 min to form the lower ionogel layer. The flexible heater is then placed on the surface of the polymerized ionogel, and the upper mold is assembled. The process is repeated to prepare the upper ionogel layer, fully encapsulating the flexible heater. The completed smart adhesive pad measures 25 mm in length, 15 mm in width, and 1.8 mm in thickness. The optical and infrared images of the pad are shown in Figure 4b. Figure 4c demonstrates the excellent compliance of the smart adhesive pad under bending and twisting, indicating its potential for conformal integration onto the curved surfaces of glove fingers.

We investigated the transient temperature distribution of the smart adhesive pad under different direct current input voltages and after power‐off at an ambient temperature of 25 °C, using an infrared (IR) camera (RSE60, Fluke, USA) and an in situ temperature sensor (Figure 4d). The IR camera measured the average surface temperature of the pad. The results showed a good agreement between the temperature responses of the in situ sensor and the IR camera, indicating the sensor's ability to provide real‐time, precise temperature monitoring. Notably, the addition of thermally conductive (TC) silicone around the in situ temperature sensor significantly improved its temperature response speed during rapid heating (Figure 4d; Figure S10, Supporting Information). During the heating process from room temperature to 45 °C under different voltages, the maximum temperature error between the sensor with TC silicone and the IR camera was markedly reduced compared to the sensor without TC silicone (Figure 4e). The sensor without TC silicone exhibited maximum temperature errors of 2.1, 6.4, 8.7, and 9.9 °C at 1, 2, 3, and 4 V, respectively. In contrast, the sensor with TC silicone showed significantly lower errors of 1.4, 2.2, 3.3, and 4.0 °C at the same voltage levels. Furthermore, Figure 4f illustrates the relationship between the heating time and the input voltage for the smart adhesive pad. The time required to heat the pad from 25 to 45 °C decreased as the input voltage increased, with the heating time reduced to 4 s at 4 V. However, the cooling process was relatively slow, taking 510 s to cool from 45 to 25 °C. For the thermoresponsive ionogel with switchable adhesion, precise temperature control is critical for regulating the adhesion strength. By leveraging real‐time temperature feedback from the in situ sensor and adjusting the heating power through a proportional‐integral‐derivative (PID) control system (Figure 4h), the pad was able to achieve and maintain four target temperatures (30, 35, 40, and 45 °C) with 5 °C intervals during both heating and cooling processes at an ambient temperature of 25 °C (Figure 4g). This experiment demonstrates that the smart adhesive pad enables precise temperature control, allowing automatic transitions between desired temperatures. As shown in Figure S11a,b (Supporting Information), the PID control system demonstrated effective closed‐loop temperature control capabilities at both lower (15 °C) and higher (35 °C) ambient temperatures. Besides, the smart adhesive pad exhibited a rapid response to temperature changes between 15 and 35 °C, consistently maintaining a target temperature of 45 °C (Figure S11c, Supporting Information). The experimental results showed that the PID control system demonstrated good adaptability to different ambient temperatures and robust performance in response to rapid ambient temperature changes.

### 2.4 The Hands‐Free Control Interface Based on IMU

While performing a primary task, humans can still move other body parts without interfering with that task's execution. The inherent redundancy and flexibility of the human body can be used to control wearable robotic systems.^[^ ^38^, ^39^ ^]^ In this study, we use little finger movement as control input and design a hands‐free control interface based on IMU, enabling users to achieve easy and intuitive detachment control. There are two primary reasons for considering little finger motion. First, the little finger typically plays a supportive role in object grasping. Among all fingers, the thumb contributes 40% to hand function, while both the index and middle fingers contribute 20% each, and the ring and little fingers account for 10% each.^[^ ^40^ ^]^ Second, the little finger exhibits a high degree of independence, surpassed only by the thumb and index fingers, demonstrating minimal motion coupling with the other fingers.^[^ ^41^, ^42^ ^]^ For patients with rheumatic diseases who are unable to move the little finger, the movement of another functional finger may be employed as a control input, serving as a backup solution.

**Figure 5:**
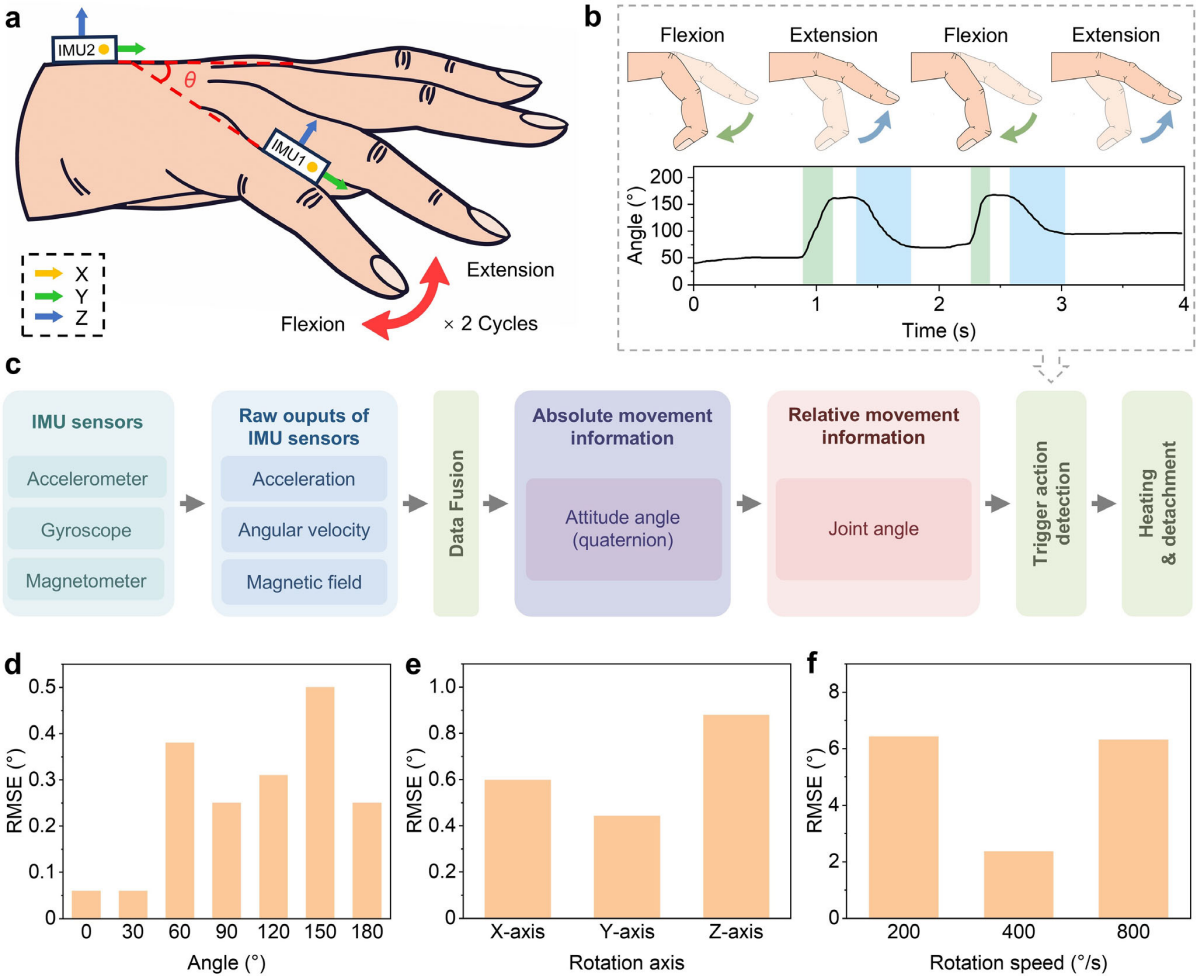
Hand release intention detection via the hands‐free control interface based on IMU. a) IMU orientation and joint angle of the little finger. b) Detachment trigger mechanism. Detachment is triggered by two consecutive extensions and flexions of the little finger. c) Schematic flow diagram of data processing steps for detecting the user intention. d) RMSE between the IMUs estimated angles and the ground truth static angles. e) RMSE between the reference and measured angles under conditions where both IMUs were fixed at a 90° angle and rotated about the x, y, and z axes at an angular velocity of 100°/s. f) RMSE between the reference angles provided by the servo motor and those measured by the IMUs at rotation speeds of 200, 400, and 800°/s.

We integrated a nine‐axis IMU sensor (BNO055, Bosch Sensortec, Germany) on the FPCB located on the back of the hand, and affixed a finger‐mounted IMU module (Figure S12, Supporting Information) to the little finger using a 3D‐printed flexible adapter. This dual‐IMU setup allows for the monitoring of the little finger joint movements (**Figure** **5**a). The U‐shaped adapter is fabricated from thermoplastic polyurethane using 3D printing technology; one flat side is adhered to the IMU with double‐sided tape, while the opposing side is attached to the finger (Figure S13, Supporting Information). The absence of a smart adhesive pad on the little finger allows free flexion and extension. Two consecutive flexion and extension movements of the little finger are interpreted as the user's intention to release an object (Figure 5b). Upon detection of the corresponding motion signals by the IMUs, the flexible heater within the smart adhesive pad activates. As the ionogel's temperature rises, adhesion strength decreases, enabling easy detachment. To prevent potential thermal injury, the heating stops automatically when the in situ temperature sensor detects a rise to 45 °C. Notably, this detachment control system enables hands‐free operation, removing the need for physical interaction with mechanical switches, buttons, or graphical user interface devices using the other hand, thereby allowing for collaborative task execution with both hands (Table S1, Supporting Information).

The flowchart of the hand release intention detection algorithm is depicted in Figure 5c. The BNO055 IMU integrates a three‐axis accelerometer, gyroscope, and magnetometer, enabling the measurement of three‐axis acceleration, angular velocity, and magnetic fields. Employing its built‐in data fusion algorithm, the BNO055 outputs the absolute orientation in the form of quaternions. Once the absolute orientation angles for each IMU are acquired, the relative orientation angle between the two IMUs can be calculated. Considering that IMU 1 on the little finger and IMU 2 on the back of the hand are aligned parallel along the x‐axis, the roll angle of the relative orientation angle in Euler form corresponds to the joint angle of the little finger. In this study, IMU 1 is positioned on the middle phalanx of the little finger, thus the computed joint angle *θ* represents the sum of the metacarpophalangeal and proximal interphalangeal joint angles. Real‐time angle signals are collected at an approximate frequency of 100 Hz. A window with a width of 50 sampling periods is applied to detect flexion or extension of the little finger using a threshold‐based method. A flexion motion is identified if the minimum angle occurs before the maximum angle within the window and their difference exceeds the threshold. Conversely, if the maximum angle precedes the minimum angle and the difference also exceeds the threshold, an extension motion is determined. Heating is triggered upon detecting sequential flexion‐extension‐flexion‐extension movements. The interval between flexion and extension movements must not exceed 100 sampling periods, else the previous movement state information is cleared.

To validate the accuracy of the joint angle measurement algorithm, static and dynamic tests were conducted. In the static test, two IMUs were mounted on a 3D‐printed model at fixed angles of 0°, 30°, 60°, 90°, 120°, 150°, and 180°, with data collected for 5 min at each angle. These angles were selected as they evenly divide the accessible range of the joint angle *θ*. The IMU‐measured angle data are presented in Figure S14 (Supporting Information), and the root mean square errors (RMSEs) with respect to static reference angles are shown in Figure 5d. All RMSEs were below 0.5°, indicating good static accuracy of the measurement algorithm. A servo motor (STS3025BL, Feetech, China) was used for the dynamic testing platform. Dynamic tests included two experiments, with the schematic diagram of the experimental setup shown in Figure S15 (Supporting Information). In the first, both IMUs were fixed at a 90° angle on the servo motor's moving arm, rotating around the x, y, and z axes of IMU 2 within a range of ‐90° to 90° at an angular velocity of 100°/s. This setup simulates hand movement without the little finger's flexion and extension. The angle measurements are provided in Figure S16 (Supporting Information), with corresponding RMSEs shown in Figure 5e: 0.60° for the x‐axis, 0.44° for the y‐axis, and 0.88° for the z‐axis. These results indicate that the algorithm is nearly unaffected by hand movements. Subsequently, the IMUs were respectively attached to the moving arm and fixed support of the servo motor, which performed reciprocal rotations within a ‐90° to 90° range at angular velocities of 200°/s, 400°/s, and 800°/s to simulate finger flexion and extension. Simultaneously, the reference angles provided by the servo motor and those measured by the IMUs were recorded, as shown in Figure S17 (Supporting Information). Figure 5f presents RMSEs between reference and measured angles at different speeds: 6.43°, 2.36°, and 6.31°, which are comparable to the RMSE values reported in other studies,^[^ ^43^, ^44^, ^45^, ^46^ ^]^ ranging from 1.45° to 4.18°. These results demonstrate acceptable dynamic accuracy of the measurement algorithm under high‐speed conditions. Overall, the static and dynamic test results demonstrate that the joint angle measurement algorithm is effective under both conditions, making it suitable for hand release intention detection.

Five healthy subjects (three males and two females) were recruited to assess the robustness of the hand release intention detection algorithm. Initially, the subjects underwent a preliminary assessment to establish their personalized thresholds. Each subject donned the glove and performed 10 triggering actions—specifically, two consecutive flexion and extension movements of the little finger—in both palm‐horizontal and palm‐vertical orientations, with angle signals recorded by two IMUs. The angle threshold initiated at 1° and increased in increments of 1°. The collected angle signals were subsequently analyzed to ascertain whether this threshold effectively identified all triggering actions. The midpoint of the threshold range that successfully recognized all triggering actions was defined as the personalized threshold for each subject. Thereafter, each subject's personalized threshold served as the test threshold for the algorithm, and the success rate of 50 triggering attempts was evaluated for each subject while wearing the glove in both palm‐horizontal and palm‐vertical orientations. When the five subjects employed their personalized thresholds (21°, 14°, 20°, 37°, and 24°), the triggering success rates reached 100% for all subjects. The experimental results suggest that the hand release intention detection algorithm demonstrates robust performance and high adaptability across different users.

### 2.5 Demonstrations of the Wearable Adhesive Glove

We recruited a patient with rheumatic disease (female, age 50) to evaluate the assistance performance of the wearable adhesive glove. The subject suffers from EGPA, resulting in peripheral neuropathy that affects both motor and sensory nerves, leading to hand dysfunction and limb paralysis. Specifically, her right hand exhibits motor and sensory impairments, including reduced muscle strength, impaired fine motor skills, and sensory loss. Additionally, the interphalangeal joint of her right thumb exhibits deformity and some spasticity, preventing active flexion. The subject provided written informed consent, and all experimental procedures were approved by the Biomedical Ethics Committee of Beihang University (Approval No. BM20240284). During the experiments, the subject's personalized threshold for the hand release intention detection algorithm was set to 25°.

**Figure 6:**
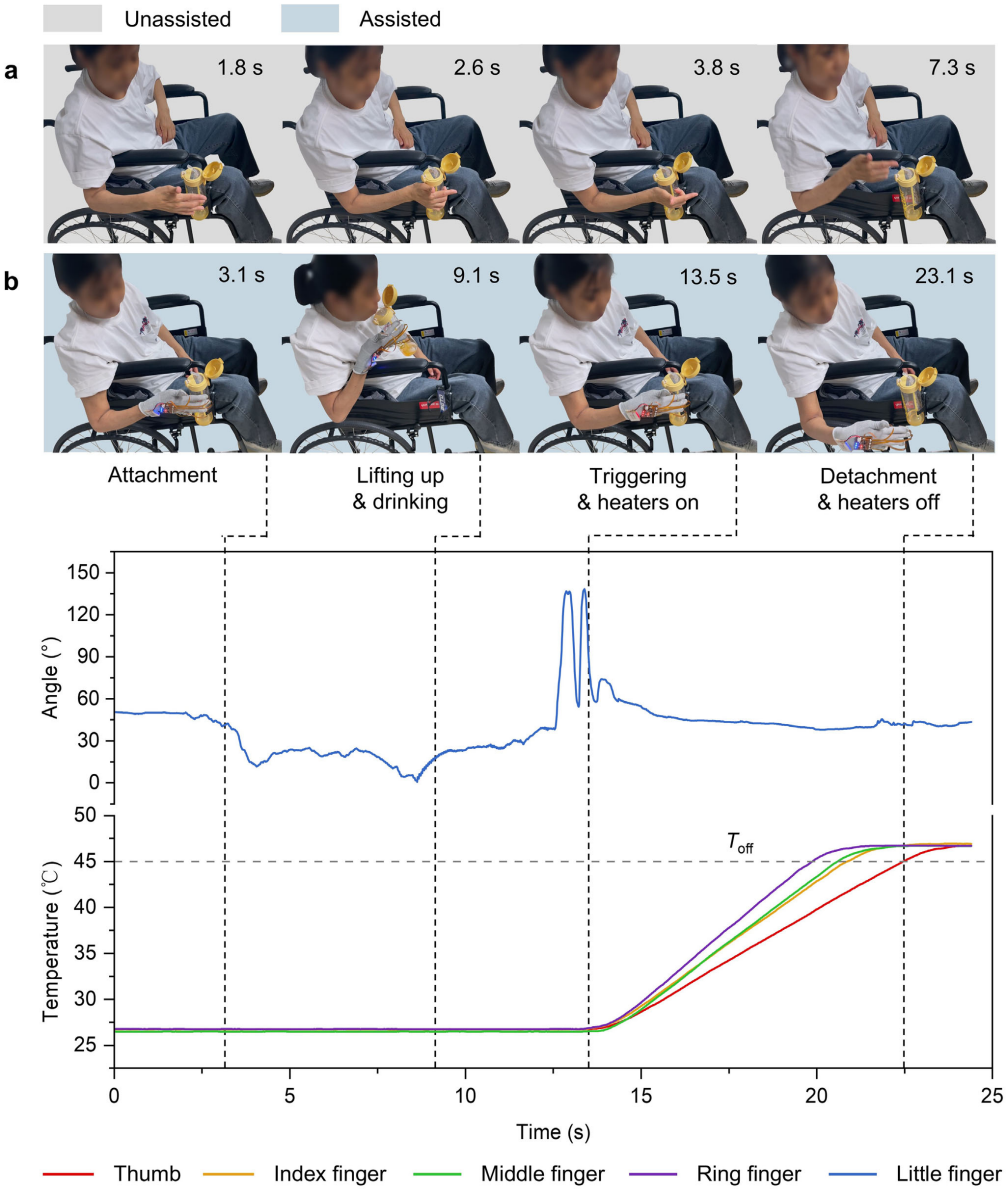
Example of performance of the drinking task performed by the rheumatic patient without and with assistance of the glove. a) Selected snapshot images showing the patient was not able to pick up the water bottle without the assistance of the glove. b) Demonstration of the patient to grip, lift, drink from, and release the water bottle with the assistance of the glove. The curves below show real‐time measurements of the little finger's angle and the temperature of other finger's smart adhesive pads during this process.

To evaluate the feasibility of using the wearable adhesive glove to assist rheumatic patients with hand dysfunction in grasping tasks, we performed tests both with and without the glove's assistance during a drinking task (**Figure** **6**; and Movie S1, Supporting Information). The subject was asked to pick up a bottle containing a small amount of juice from a wheelchair's bottle holder, drink from it through a straw, and then return the bottle. Without the glove, the subject was unable to lift the bottle after four attempts. However, with the glove, the task was completed in 24 s. Figure 6b provides snapshots of the successful task completion with the glove, displaying changes in the joint angle of the little finger recorded by the IMU and the temperature of the smart adhesive pads on other fingers as measured by temperature sensors. Initially, the subject made contact with the bottle and applied preload with the glove. The high adhesion strength of the ionogel at room temperature enabled the smart adhesive pads to assist in lifting the bottle for drinking. The subject then returned the bottle and executed two consecutive flexion and extension movements of the little finger to activate the detachment of the smart adhesive pads. Upon detecting the detachment trigger signals, the pads' flexible heaters warmed the ionogel until it reached a threshold of 45 °C, stopping the heating process. Despite stopping, thermal inertia caused the temperature to rise, peaking at ≈46.7 °C. The rising temperature led to reduced adhesion strength, easing the glove's detachment from the bottle. The results demonstrate that the proposed glove provides effective assistive grasping capabilities by accurately controlling four smart adhesive pads based on the measured motion of the little finger. During the heating and cooling process, the highest temperature recorded on the inner surface of the glove was 38.8 °C (Figure S18, Supporting Information), which is significantly below the threshold for low‐temperature burns (45 °C), thereby ensuring safety for wearable applications.

**Figure 7:**
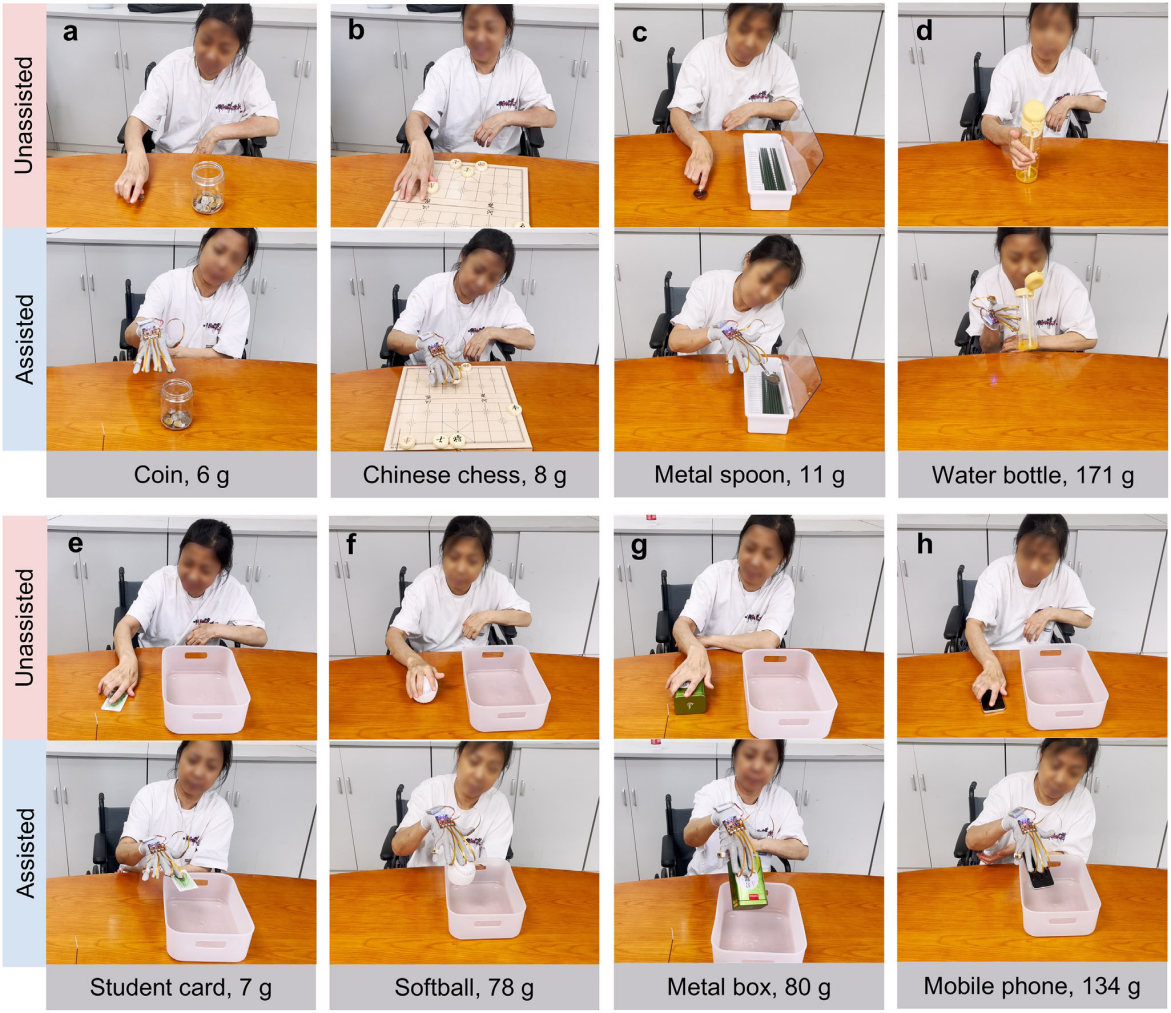
Different simulated ADL tasks performed by the patient with rheumatic disease for unassisted (without glove) and assisted (with glove) conditions. a) Picking up a coin and placing it into a money jar. b) Playing Chinese chess to kill the opponent. c) Picking up a metal spoon and placing it into a cutlery organizer. d) Grasping the bottle, drinking juice from the bottle through a straw, and placing it back on the table. Grasping various objects of different shapes and sizes and placing them in the storage box: e) student card, f) softball, g) metal box, h) mobile phone. Without the assistance of the glove, the subject was not able to complete the tasks, whereas with the glove's assistance, the subject could complete the tasks.

To further validate the efficacy of the glove, the subject was asked to perform a series of simulated ADL tasks both with and without the glove. These tasks included placing a coin into a money jar, achieving checkmate in Chinese chess, placing a metal spoon into a cutlery organizer, lifting and setting down a bottle after drinking, and placing items such as a student card, softball, metal box, and mobile phone into a storage bin (**Figure** **7**; and Movie S2, Supporting Information). Without the glove, the subject was unable to complete any tasks. However, with the glove, all tasks were successfully completed with an average completion time of 16.4 s. For small and light objects like the coin, Chinese chess piece, metal spoon, and student card, a single smart adhesive pad was sufficient. In contrast, larger items such as the bottle, softball, metal box, and mobile phone required multiple smart adhesive pads. These results highlight the glove's adaptability to various objects of different shapes, sizes, materials, and weights, affirming its role as an effective assistive device for patients with rheumatic diseases.

In addition, we investigated the power consumption of the wearable adhesive glove system. The experimental results demonstrated that the system sustained an average power consumption of 0.13 W during standby. Heating a single smart adhesive pad caused the power consumption to rise to an average of 9.6 W. When four smart adhesive pads were heated simultaneously, the power consumption increased to 24.1 W. Additionally, we tested the heating‐detachment cycles supported by a 1200 mAh battery powering the wearable adhesive glove at various ambient temperatures. At ambient temperatures of 15, 25, and 35 °C, the battery supported ≈200, 280, and 512 cycles for single‐finger heating‐detachment, as well as 50, 70, and 127 cycles for four‐finger heating‐detachment, respectively. In the future, the device's power consumption can be optimized to enhance its operational duration. One potential solution involves designing slimmer smart adhesive pads to reduce the energy required for heating. Another approach is to integrate capacitive sensors^[^ ^47^ ^]^ or optical proximity sensors^[^ ^32^ ^]^ into the smart adhesive pads to detect contact with objects, which enables targeted heating of only the specific fingers making contact during detachment.

## 3 Discussion and Conclusion

In this work, we present a portable grasp‐assist glove with soft switchable adhesives, designed to aid patients suffering from rheumatic diseases. This device features controllable adhesion that can be switched according to user intent. The smart adhesive pads switch from high to low adhesion within 4 s heating under a 4 V voltage. Furthermore, a hands‐free control interface based on IMU employs two consecutive flexion and extension movements of the little finger as the trigger for detachment, allowing for easy and intuitive detachment control. To evaluate the glove's efficacy, a comparative experiment was conducted with a rheumatic patient exhibiting hand function impairment. The subject performed simulated ADL grasping tasks both with and without the glove. With the glove, the subject successfully grasped eight different daily living objects with various shapes, sizes, materials, and weights. In contrast, without the glove, the subject was unable to complete any tasks. The experimental results demonstrate that the proposed glove enhances the patient's grasping capabilities and autonomy in ADL.

Unlike traditional soft wearable assistive gloves that rely on hydraulic/pneumatic or motor‐cable systems, the proposed soft wearable adhesive glove utilizes the thermoresponsive switchable adhesion property of smart ionogel. This design employs adhesion for grasping tasks, eliminating the need for bulky external hardware such as pumps, tubes, motors, and cables to drive finger movement. This enhances system portability while avoiding the risk of injury or pain from excessive driving forces on the patient's fingers. Additionally, adhesion‐based grasping simplifies the grasping task, reducing the need for precise control of finger movements. This allows the system to function without the integration of numerous sensors or complex control algorithms, thus decreasing system complexity and demonstrating physical intelligence.^[^ ^48^, ^49^ ^]^ These benefits significantly simplify the design of the soft wearable adhesive glove, leading to a substantial reduction in system weight, achieving a total weight of 47 g, which is the lightest among reported wearable assistive gloves to date. Although physical intelligence mitigates certain control demands, adhesive manipulation beyond pick‐and‐place tasks continues to pose significant challenges for the current system. This includes the coordinated control of multifinger adhesion, such as the selective heating‐detachment of specific fingers during manipulation tasks. To effectively address this issue, precise recognition of complex finger movement intentions is essential, potentially necessitating the integration of various intention recognition methods.^[^ ^7^ ^]^ Furthermore, compared to existing pneumatic wearable adhesive gloves, the proposed glove not only offers greater portability but also features hands‐free detachment control, enabling untethered operation and intelligent control of adhesive grasping. The feasibility of aiding rheumatic disease patients with impaired hand function to independently perform ADL grasping tasks through adhesion‐based grasping was validated for the first time via clinical testing. Future research may extend the application of this device to other hand function impairments, such as those resulting from stroke, spinal cord injuries, and amyotrophic lateral sclerosis.

However, there are several limitations in the current work that necessitate further improvements. First, due to the limited adhesion strength of the PBA/[EMIM][NTf_2_] ionogel, the existing wearable adhesive glove can only grasp lightweight objects. Increasing the number of smart adhesive pads on the fingers and palm to enhance the contact area could improve payload capacity. Moreover, the adhesion strength of ionogels demonstrates significant variability around room temperature (25 °C–35 °C), which could impact the device's reliability in practical applications, particularly limiting the use of wearable adhesive gloves in environments where temperature control is impractical (e.g., outdoor settings and indoor environments without air conditioning). A promising solution lies in integrating soft thermoelectric devices^[^ ^50^, ^51^, ^52^ ^]^ that offer both heating and cooling capabilities, enabling closed‐loop temperature regulation of ionogels across various environmental conditions, thereby improving the environmental adaptability of the devices. It is important to note that the conducted study presents a proof‐of‐concept demonstration for developing wearable adhesive gloves utilizing thermoresponsive soft switchable adhesives to enhance the grasping function of patients with rheumatic diseases, the design principle of which is quite universal. The PBA/[EMIM][NTf_2_] ionogel used in the prototype may be substituted in the future with other thermoresponsive materials with switchable adhesion that exhibit superior performance. Second, the natural cooling time of the smart adhesive pad is relatively long (≈510 s), which limits the frequency of continuous adhesive grasping by the wearable glove. A promising solution is the integration of soft thermoelectric devices for active cooling. Lastly, only preliminary clinical tests have been conducted to validate the assistive grasping efficacy of the wearable adhesive glove. Future research should conduct statistical analyses on a broader cohort of subjects to further evaluate the glove's clinical effectiveness.

## 4 Experimental Section

### Materials

All chemicals were used as received without any further purification. Butyl acrylate (BA, monomer) was purchased from Macklin. Ethyleneglycol dimethacrylate (EGDMA, cross‐linker) and diethoxyacetophenone (DEOP, photo‐initiator) were purchased from Aladdin Industrial Corporation. 1‐ethyl‐3‐methylimidazolium bis(trifluoromethylsulfonyl)imide (>99%) ([EMIM][NTf_2_], ionic liquid) was purchased from Lanzhou Yulu Fine Chemical Co., Ltd.

### Synthesis of Ionogels

BA (monomer, with a sequence of concentrations of 60, 65, and 70 wt.%), EGDMA (0.2 mol.% relative to the monomer), and DEOP (0.1 wt.% relative to the monomer) were added to the ionic liquid, then the mixture was stirred for 10 min to obtain a homogeneous and fully transparent solution. This solution was then poured into a PTFE mold and covered with a glass slide. The polymerization was initiated by UV irradiation, and the solution was cured under UV light (365 nm, 800 mW cm^−2^) for 10 min using a UV‐LED curing device (SZUV‐II, Height‐LED, China).

### Characterization of the LCST of Ionogels

The LCST of the ionogels was determined by measuring optical transmittance at 658 nm using dynamic light scattering (Litesizer 500, Anton Paar, Austria) with a heating rate of ≈2 °C min^−1^. The ionogels were cut into rectangular shapes (20 mm × 8 mm × 1 mm) and placed along the inner wall of a quartz cuvette. A transmittance of 100% indicated homogeneity of the ionogel, whereas a decrease in transmittance suggested that the ionogels were undergoing LCST phase separation. The LCST was defined as the temperature at which the transmittance decreased to 80%.

### Characterization of Adhesion Strength

The adhesion strength was characterized by the pull‐off test, as illustrated in Figure S4 (Supporting Information). The substrate was attached to the end of the linear motor actuator (E1100, LinMot, Switzerland) using a nano double‐sided adhesive tape (33 601, Deli, China). An ionogel disc (2 mm thick, 15 mm in diameter) was fixed to a temperature control platform with silicone adhesive (SilPoxy, Smooth‐On, USA). The temperature control platform was then mounted onto a commercial high‐precision force sensor (Mini40, ATI Industrial Automation, USA) via a 3D‐printed connector. For temperature control above 25 °C, a temperature‐controlled heating plate (JK‐002, Jiukou, China) was utilized. For temperatures below 25 °C, a custom‐designed active cooling system was employed. This active cooling system consisted of a thermoelectric device (TEC1‐12702K34, Xinghe Electron, China), a water cooling circulation system, and a temperature controller (XH‐W1510, Xinghe Electron, China) for the thermoelectric device. During the measurement, the ionogel was preloaded against the substrate for a specified duration. The preload was regulated by adjusting the displacement at the end of the linear motor actuator. Subsequently, the linear motor actuator was actuated at a pulling speed of 10 mm min^−1^ until complete separation at the ionogel/substrate interface occurred. The adhesion strength was calculated as the peak force observed during the separation process divided by the initial cross sectional area of the ionogel.

### Heating and Cooling Speed Test

The constant voltage was applied to the flexible heater using a DC power supply (NPS605W, WANPTEK, China). During heating, an infrared camera (RSE60, Fluke, USA) was positioned directly above the smart adhesive pad and connected to a laptop via a USB cable. The temperature changes of the smart adhesive pad were recorded in the software (SmartView IR, Fluke, USA). Data were subsequently exported to determine the average temperature over time. Simultaneously, temperature readings from the in situ temperature sensor (MCP9700, Microchip Technology, USA) on the flexible heater were logged through the serial monitor in Arduino. The smart adhesive pad was heated to 45, 50, 55, and 60 °C when the voltage was 1, 2, 3, and 4 V, respectively, and the DC power supply was manually turned off to stop the heating, followed by natural cooling to room temperature.

### Closed‐Loop Temperature Control of the Smart Adhesive Pad

The target temperature for the smart adhesive pad was conveyed from the laptop to the MCU via a Bluetooth module. The MCU regulates the operational state of the flexible heater through general‐purpose input/output (GPIO) pins. The duty cycle of the PWM signal generated from the GPIO determines the power supplied to the flexible heater. The PID controller maintains the temperature of the smart adhesive pad at the target value based on the readings from an in situ temperature sensor on the flexible heater. The controller's parameters were set as follows: *k*_p_ = 25, *k*_i_ = 0.08, and *k*_d_ = 20. Ambient temperatures of 15 °C, 25 °C, and 35 °C were established using a semiconductor refrigerator (TF‐12L‐SX‐YL, Frestec, China), an indoor air conditioning system, and a vacuum drying oven (DZF‐6020A, Lichen, China), respectively. Rapid ambient temperature changes were achieved by moving the smart adhesive pad within the corresponding environments. Ambient temperature measurements were obtained using a thermocouple thermometer (UT325, UNI‐T, China).

### Fabrication of Flexible Printed Circuit Boards

Custom flexible printed circuit boards were designed using Altium Designer and fabricated by Shenzhen Jialichuang Technology Group Co., Ltd. The bill of materials includes an 8‐bit microcontroller unit (ATmega328P, Microchip, USA), a Bluetooth low‐energy (BLE) wireless transmission module (RF‐BM‐4044B4, RF‐star, China), a nine‐axis inertial measurement unit (IMU) sensor module (BNO055, Bosch Sensortec, Germany), an 8‐channel analog multiplexer/demultiplexer (74HC4051D, Nexperia, Netherlands), three low dropout regulators (CJ6206A30, Changjing, China), four N‐type metal‐oxide‐semiconductor (NMOS) transistor (AO3400, Alpha & Omega Semiconductor, USA), light‐emitting diodes (LEDs), and passive components such as resistors and capacitors. All circuit components were soldered onto a 70 µm thick polyimide substrate. The schematic diagram of the flexible printed circuit board is provided in Figure S3 (Supporting Information).

### System Power Measurement

The system's power was measured using a current and power monitor (INA 226, Texas Instruments, USA). This device measures circuit current and voltage, then calculates power as their product. Measurements were conducted with the lithium battery fully charged under three conditions: the heaters turned off, one heater activated, and four heaters activated.

### Human Subject Study

The study involving human subjects was approved by the Biomedical Ethics Committee of Beihang University (Approval No. BM20240284). A 50‐year‐old Chinese female patient with rheumatic diseases was recruited for the study after being fully informed about the research and providing written informed consent.

## Conflict of Interest

The authors declare no conflict of interest.

## Supporting information



Supporting Information

Supplemental Movie 1

Supplemental Movie 2

## Data Availability

The data that support the findings of this study are available from the corresponding author upon reasonable request.
