# Supplementary material for: Enhancing Grasping Function with a Thermoresponsive Ionogel Adhesive Glove for Patients with Rheumatic Diseases
Source: Adv Sci (Weinh). 2025 Mar 26;12(26):2414761. doi: 10.1002/advs.202414761 (PMC12245102; doi:10.1002/advs.202414761)
Supplement: Supplementary file 1 — Supporting Information [file ADVS-12-2414761-s003.docx]

Supporting Information

Enhancing grasping function with a thermoresponsive ionogel adhesive glove for patients with rheumatic diseases

Shiqiang Wang, Shiqing Liu, Jingfeng Zhang, Zhouyang Yu, Yifan Shao, Cong Zhao, Jiahong Ma, Xin Fu, Mengqi Yang, Lie Chen, Mingjie Liu*, Li Wen*

**Table S1**. Comparison between existing representative works and our soft wearable adhesive glove.


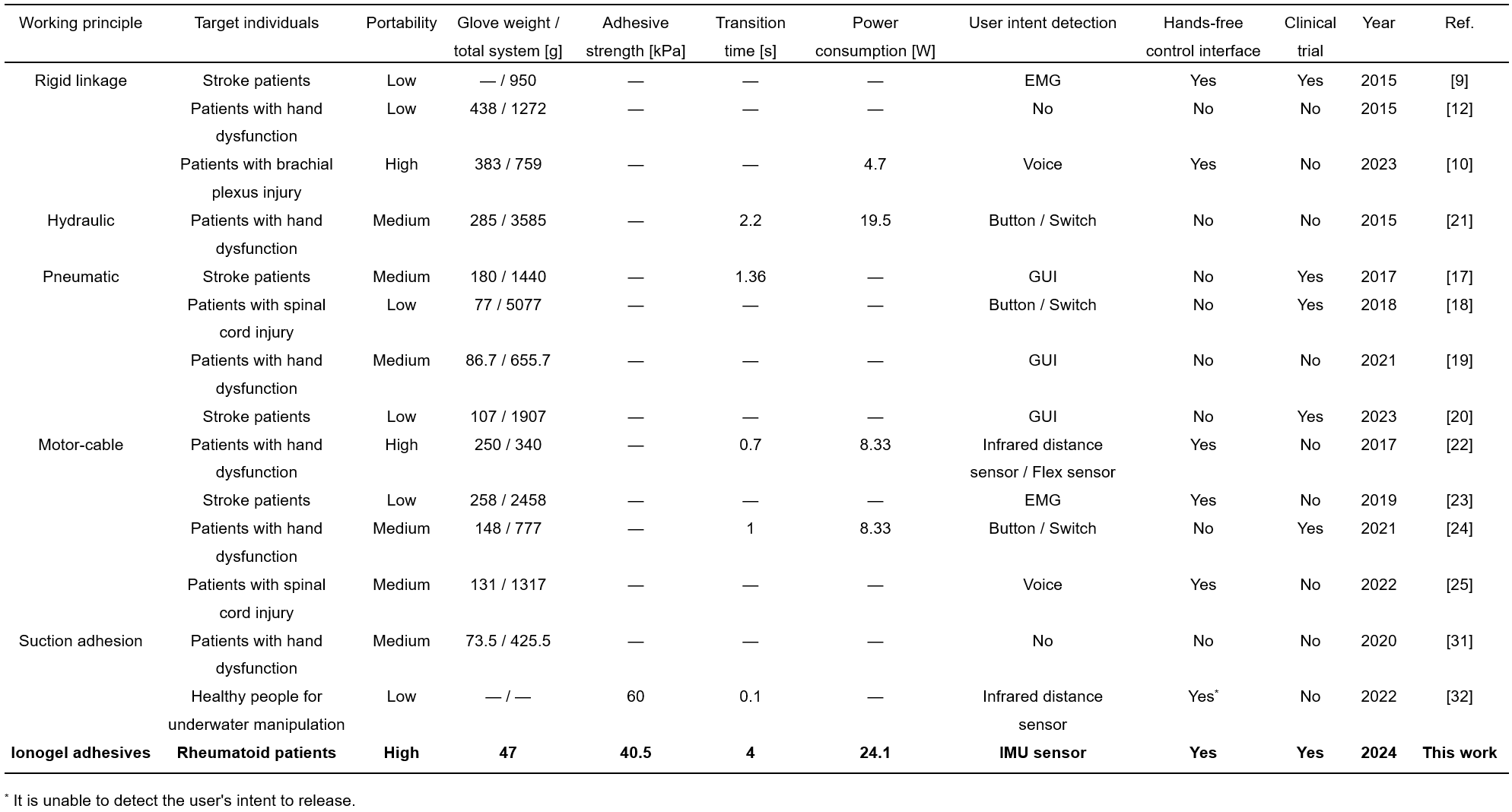


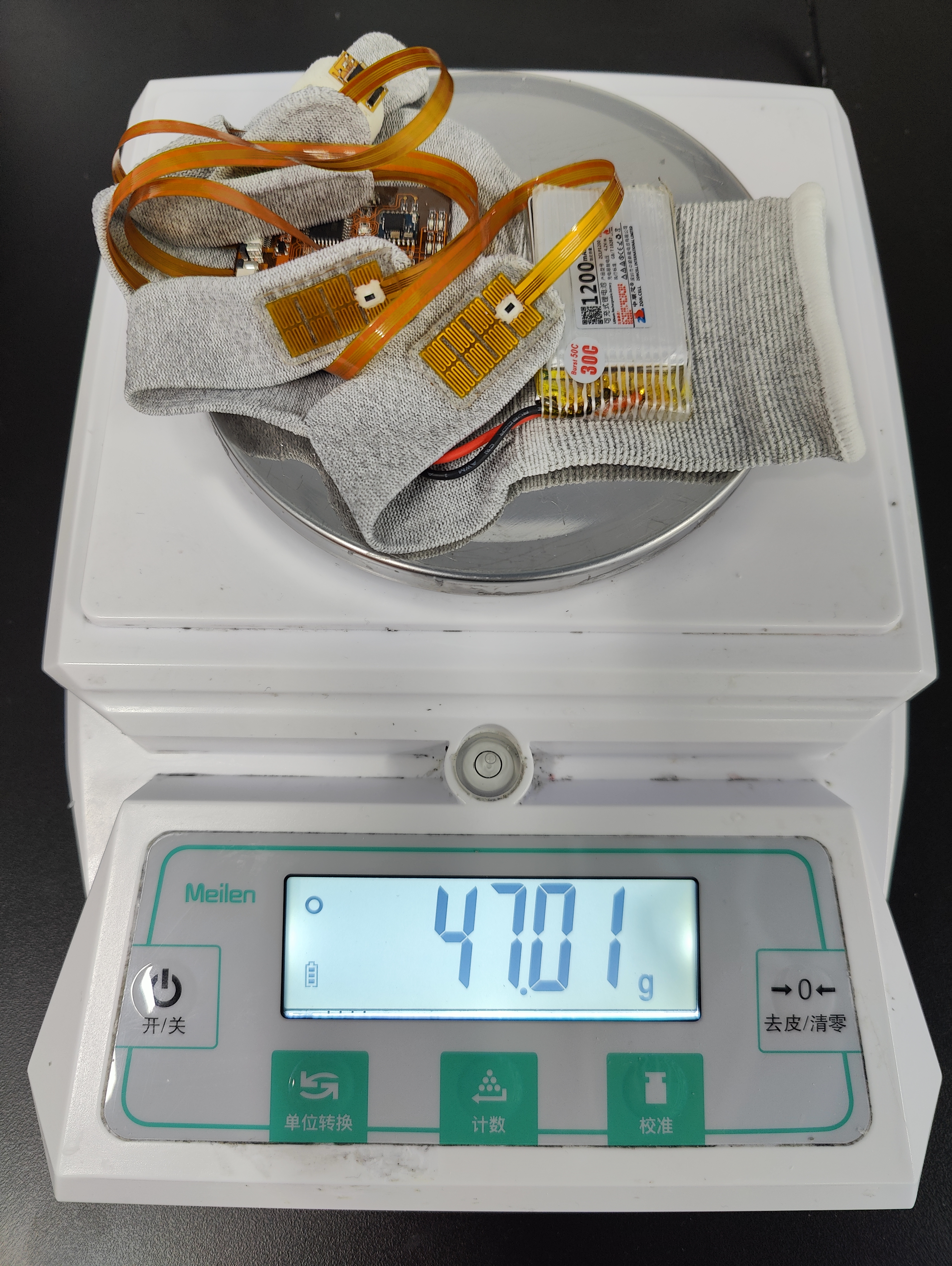


**Figure S1.** Weight measurement of the soft wearable adhesive glove.

**Table S2**. Component weight of the soft wearable adhesive glove.

| Component | Unit weight [g] | Number of components | Weight [g] |
| --- | --- | --- | --- |
| Textile glove | 8.8 | 1 | 8.8 |
| Battery | 25.6 | 1 | 25.6 |
| FPCB | 2.9 | 1 | 2.9 |
| Smart adhesive pad | 1 | 4 | 4 |
| Finger-mounted IMU module | 0.8 | 1 | 0.8 |
| Adhesives for fixing components |  |  | 4.9 |
| Total |  |  | 47 |


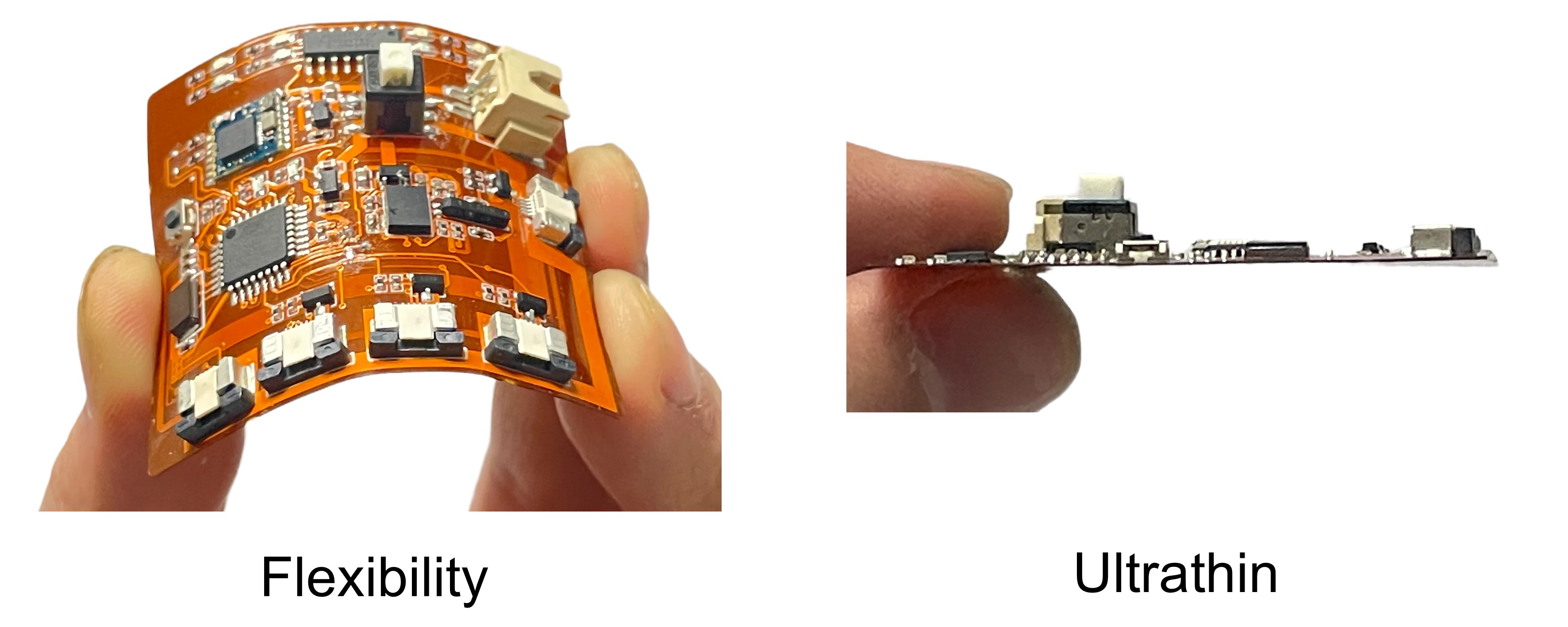


**Figure S2.** Flexible and ultrathin demonstration of the FPCB performance.


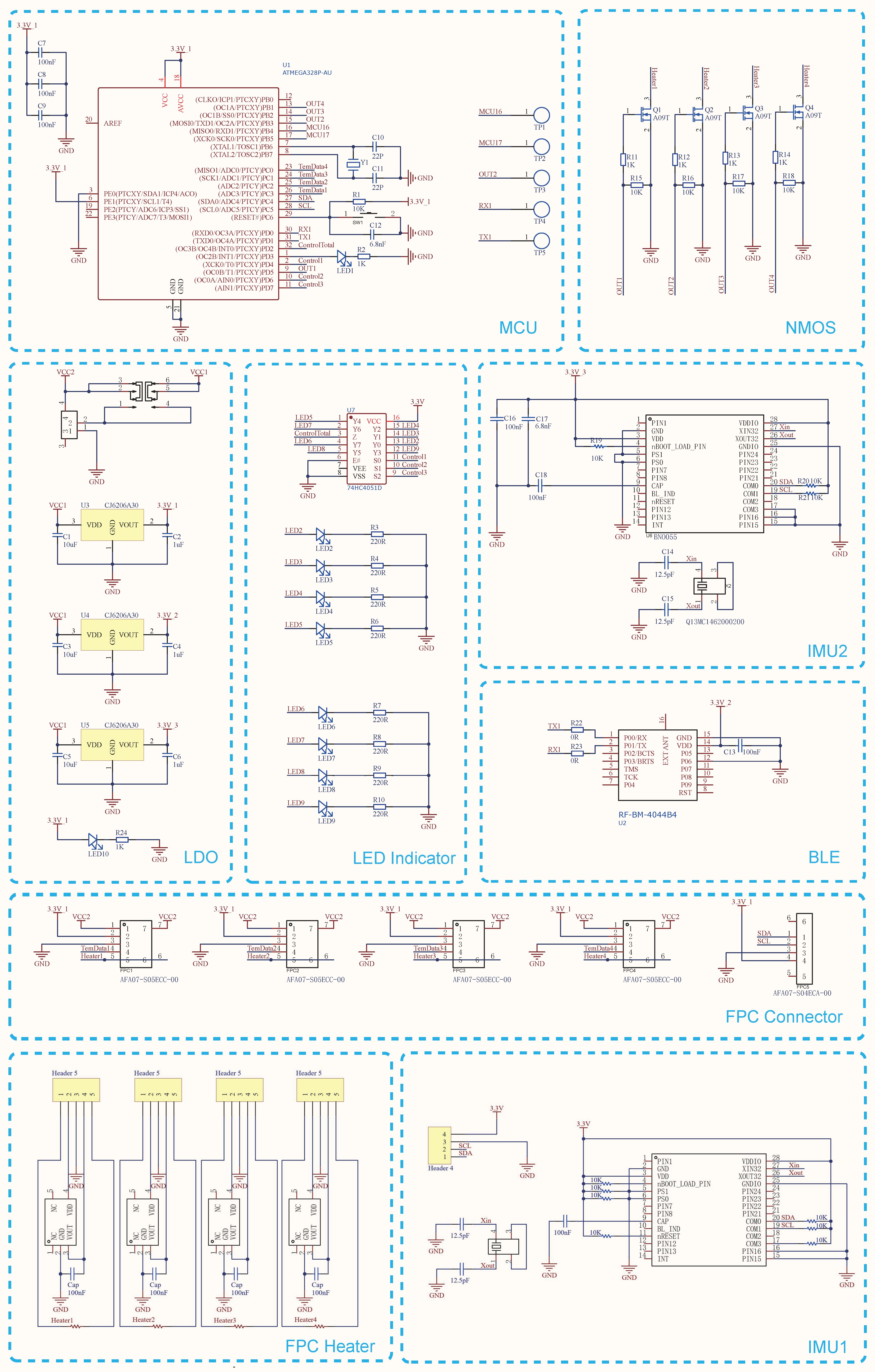


**Figure S3.** Detailed circuit schematic diagram for the integrated electronic system of the wearable adhesive glove.


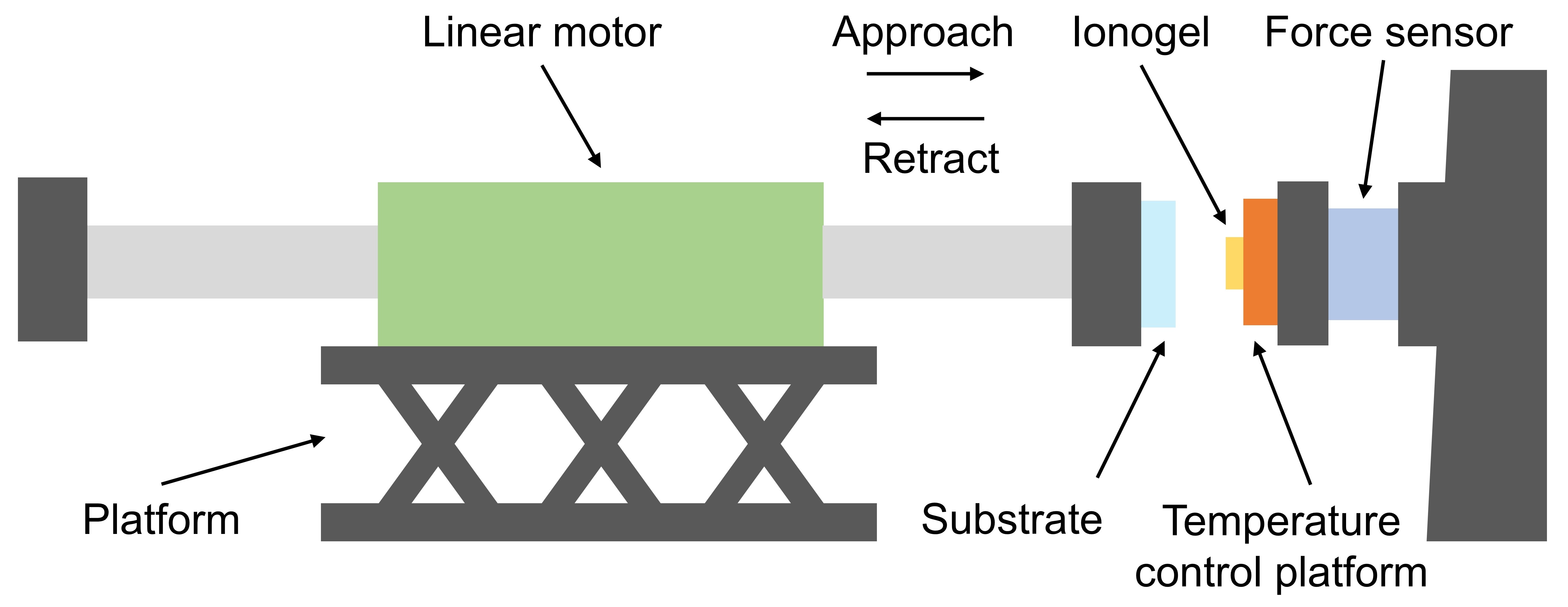


**Figure S4**. A schematic experimental setup for the adhesion measurement.

**Figure S5.** Transmittance changes of different PBA contents of ionogels with temperatures at 658 nm wavelength.


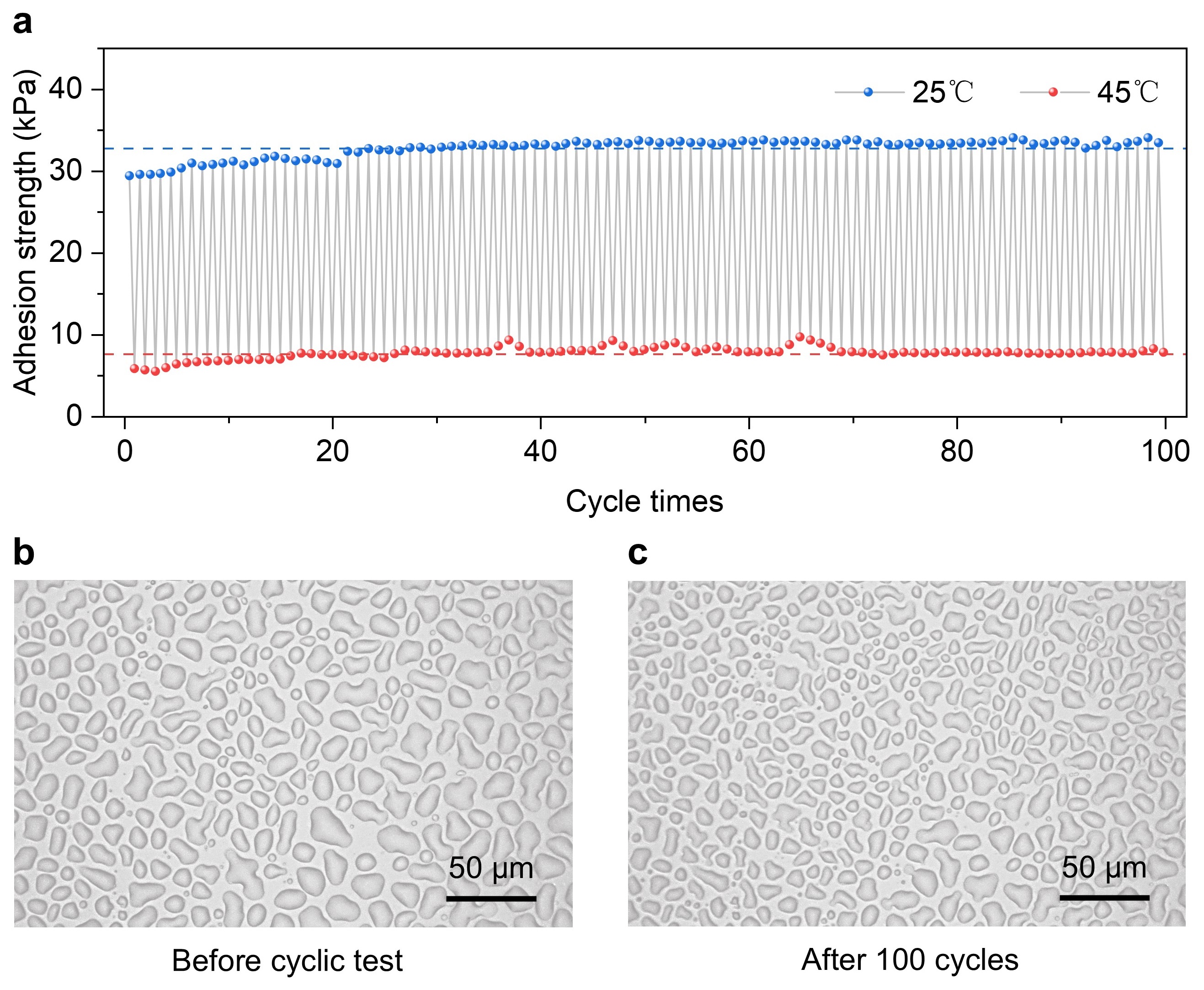


**Figure S6.** Cycling adhesion tests of the ionogels against glass substrates. a) The switchable adhesion cycles of the ionogels at 25 °C and 45 °C for 100 cycles. b) Optical microscopic image of the ionogel surface at 45 °C before and c) after the test.

**Figure S7.** Adhesion strength of ionogels under dry and wet conditions.


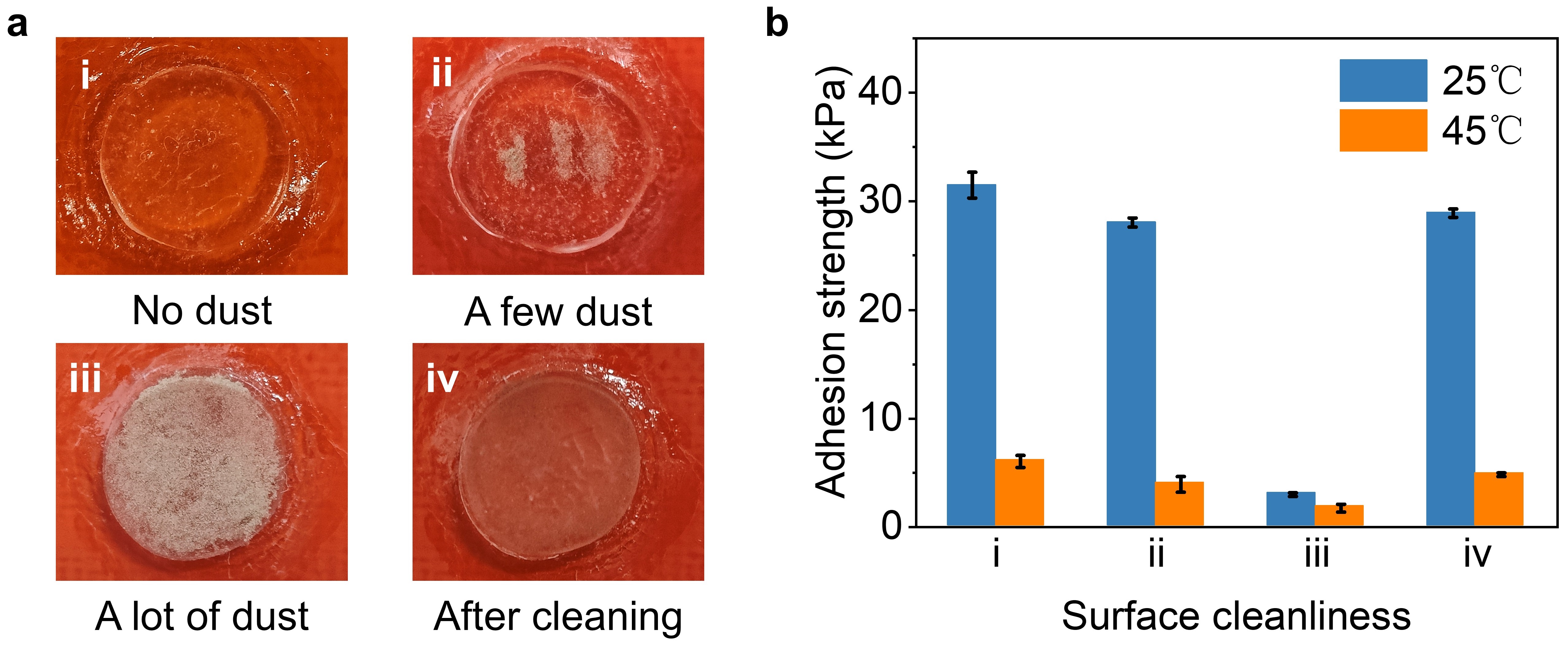


**Figure S8.** The impact of dust on the adhesion strength of ionogels. a) Optical images of the ionogel surface under the following conditions: i) no dust, ii) a few dust, iii) a lot of dust, and iv) after cleaning. b) Adhesion strength of the ionogels in the states i, ii, iii, and iv.


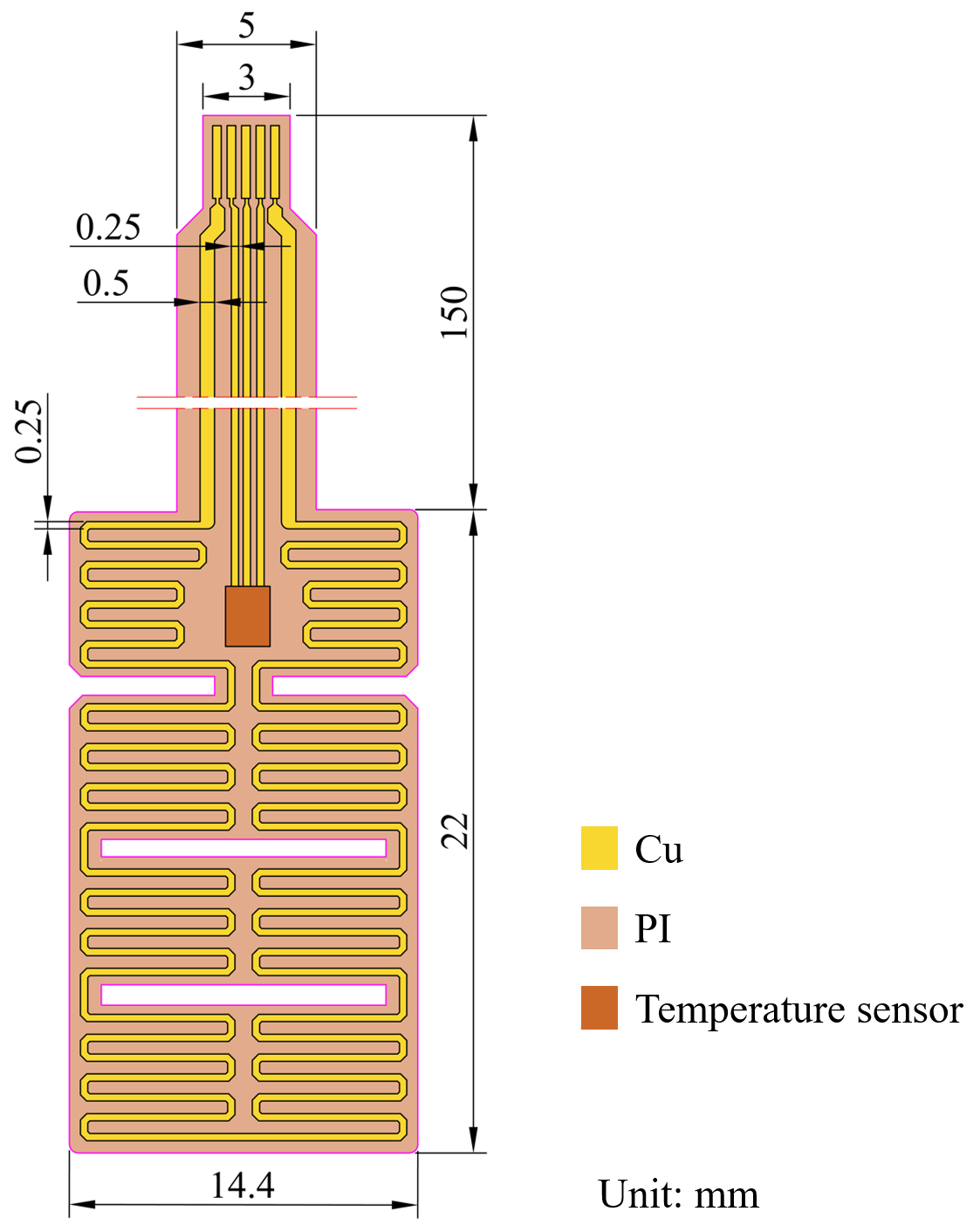


**Figure S9**. Design of the flexible heater and in situ temperature sensor.


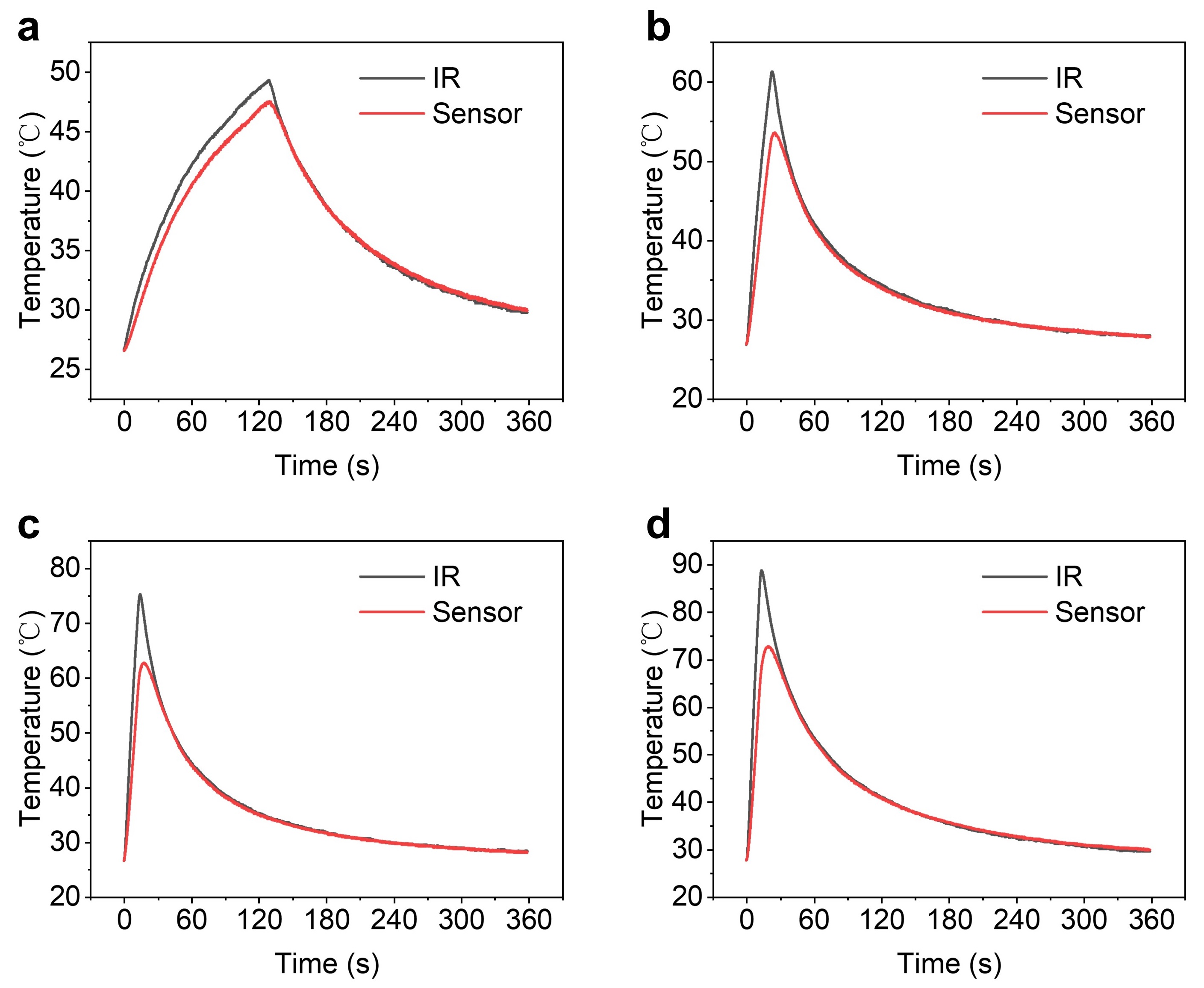


**Figure S10**. The temperature evolution of the smart adhesive pad measured via the in situ temperature sensor without TC silicone and the IR camera at different applied voltages：a) 1 V, b) 2 V, c) 3 V, and d) 4 V.


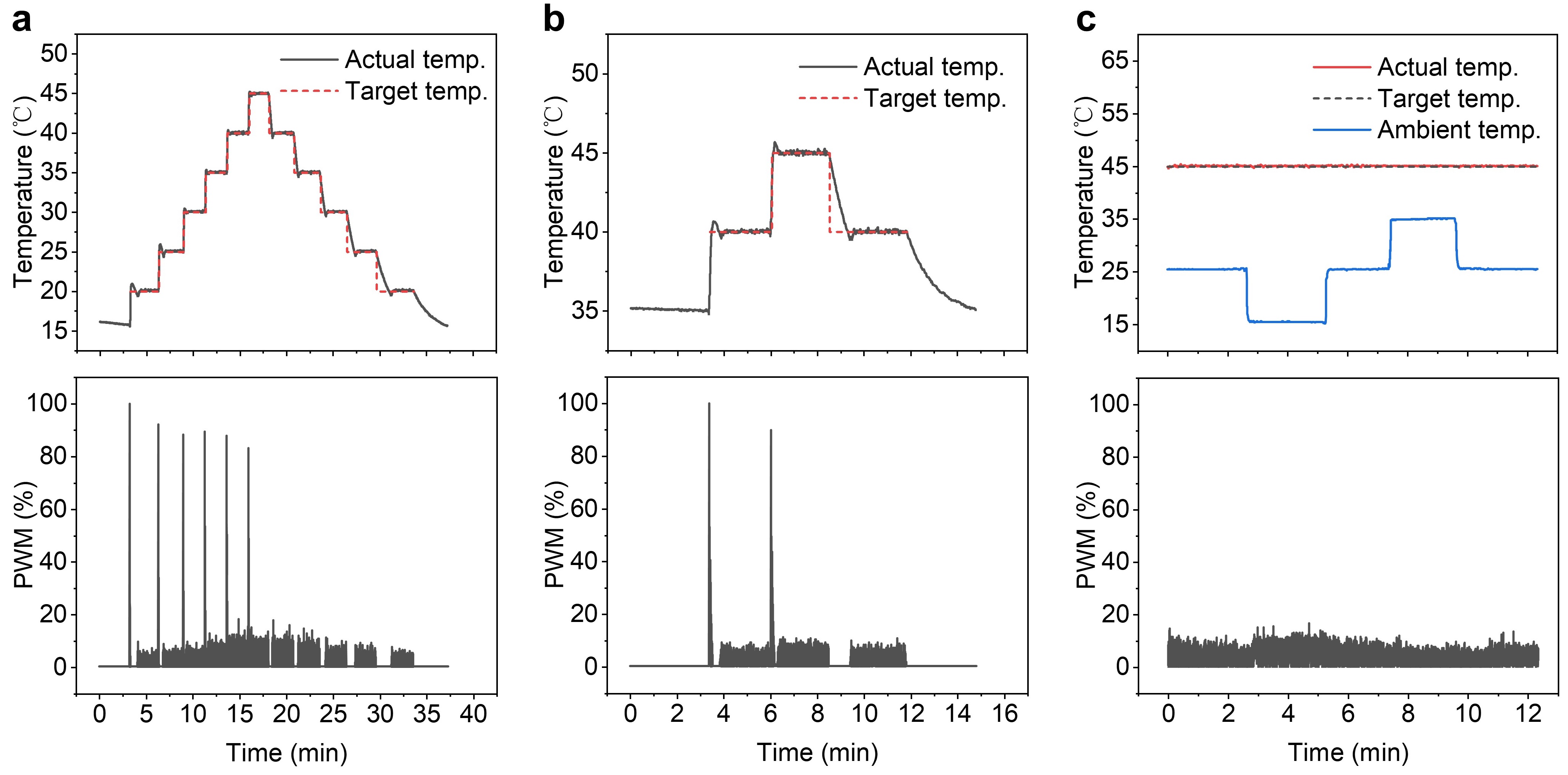


**Figure S11.** The temperature profiles and PWM values of the smart adhesive pad during temperature control performance testings. a) Temperature control performance of the smart adhesive pad at low ambient temperature (15 ℃) and b) high ambient temperature (35 ℃). c) Temperature control performance of the smart adhesive pad in response to rapid ambient temperature changes.


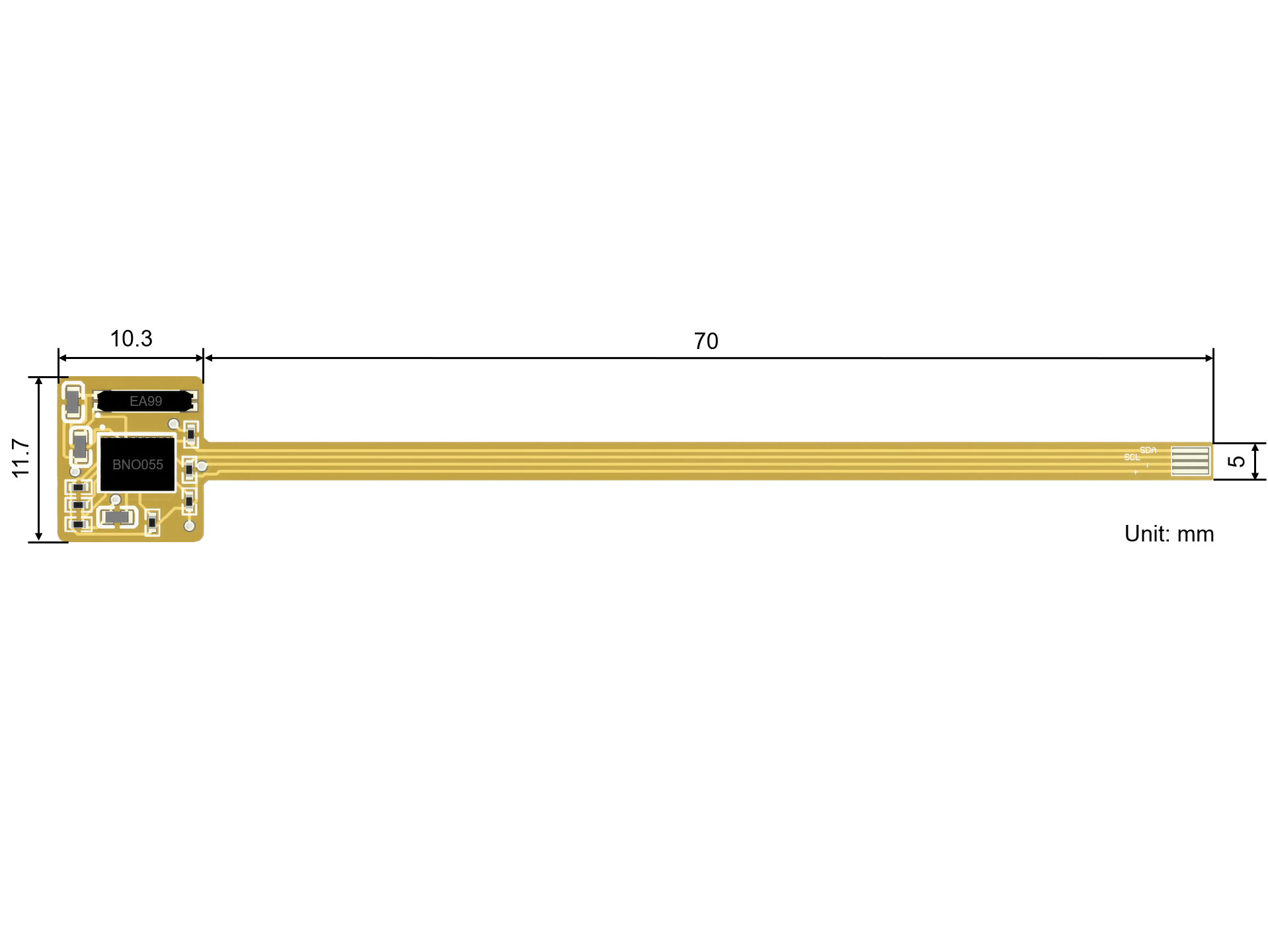


**Figure S12.** Design of the finger-mounted IMU module.


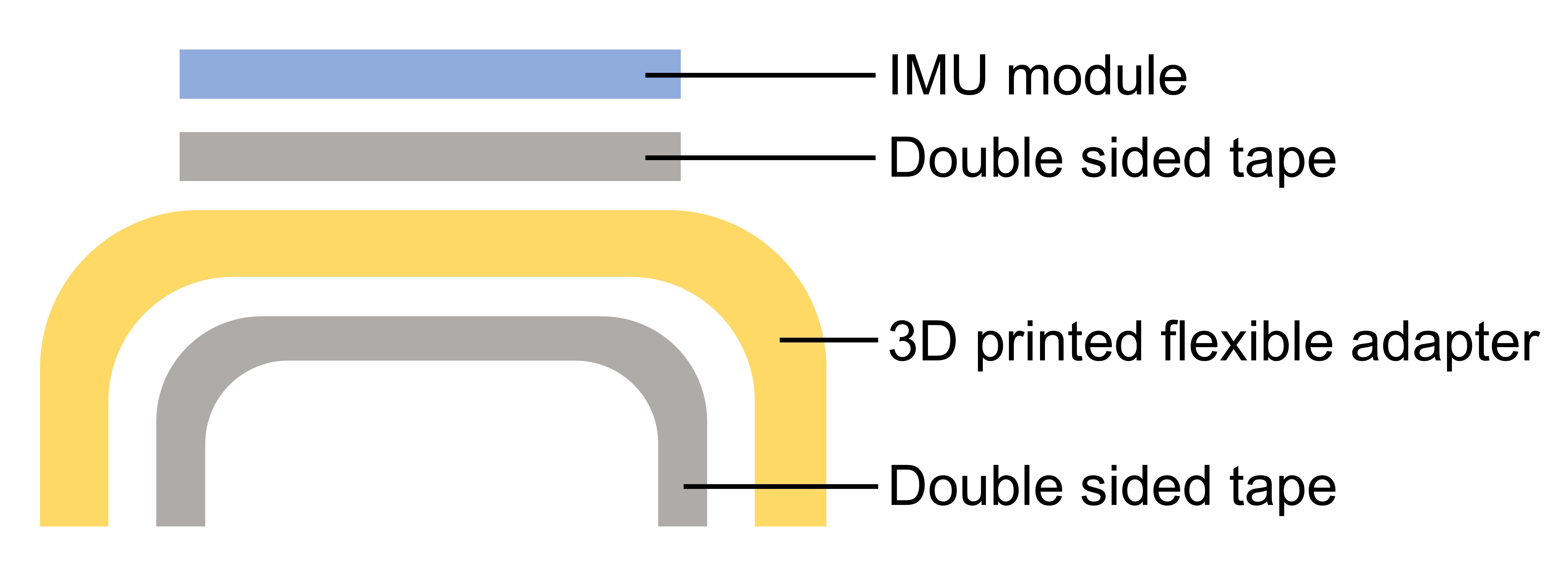


**Figure S13**. Explosion diagram of IMU finger sensor module cross-section.

**Figure S14.** Static angle measurements for 5 min.


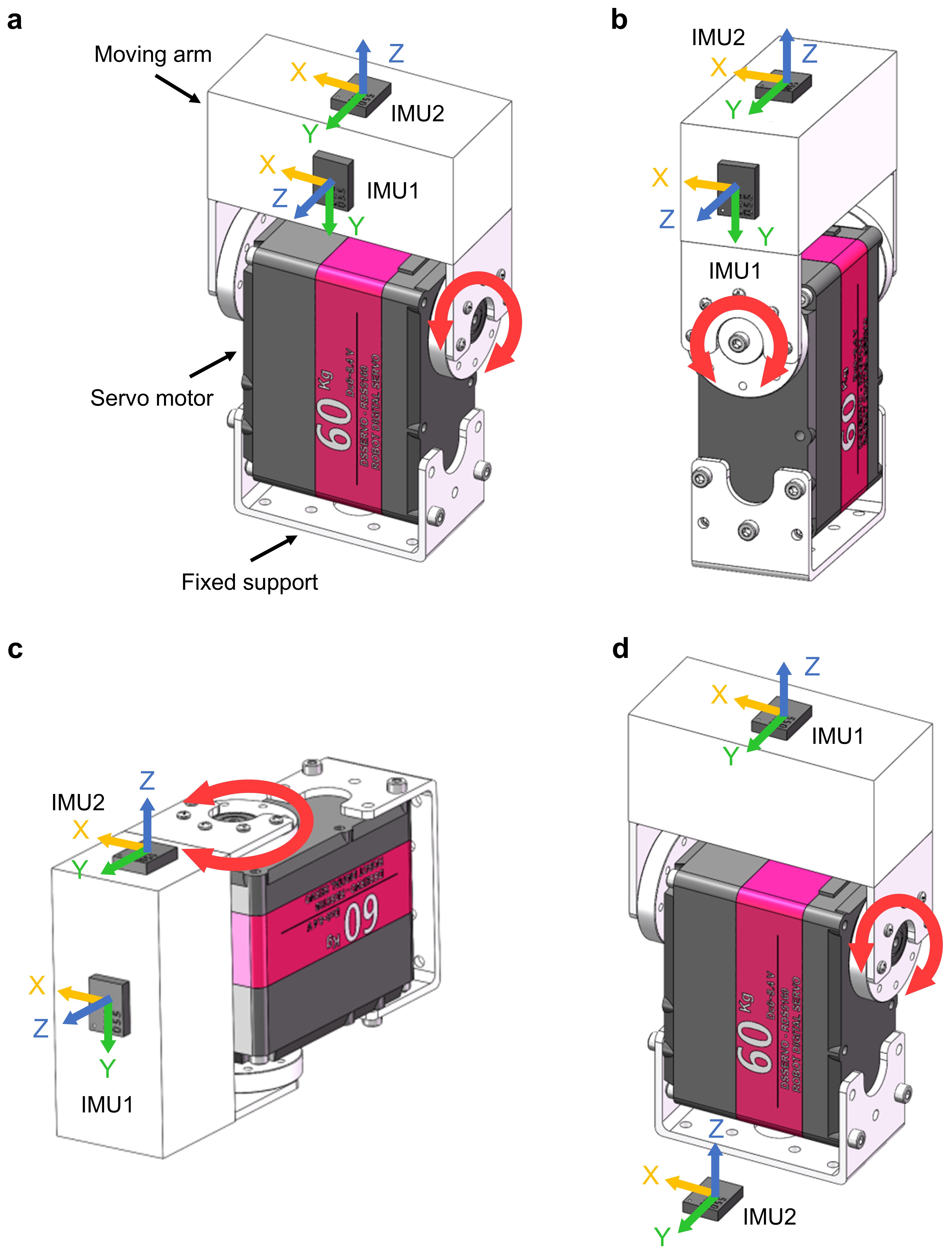


**Figure S15.** Schematic diagram of the experimental setup for the IMU dynamic tests. a) Schematic diagram of the experimental setup simulating hand movements along the x-axis, b) y-axis, and c) z-axis when the little finger is not engaged in flexion or extension movements. d) Schematic diagram of the experimental setup simulating flexion and extension movements of the little finger at varying velocities.


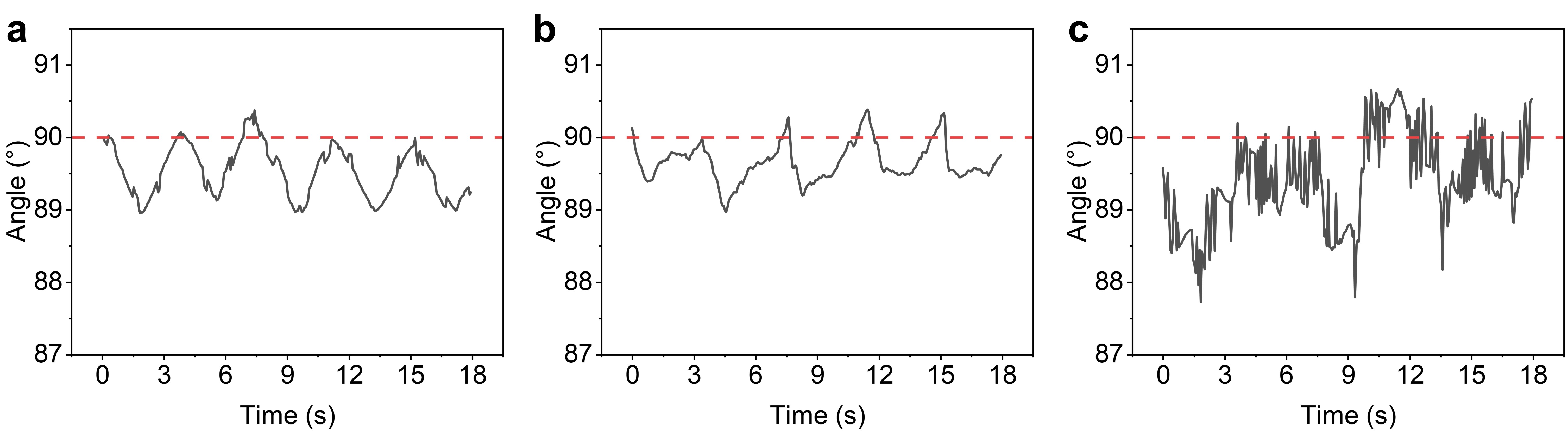


**Figure S16.** The measured angles under conditions where both IMUs were fixed at a 90° angle and rotated about the x, y, and z axes at an angular velocity of 100°/s.


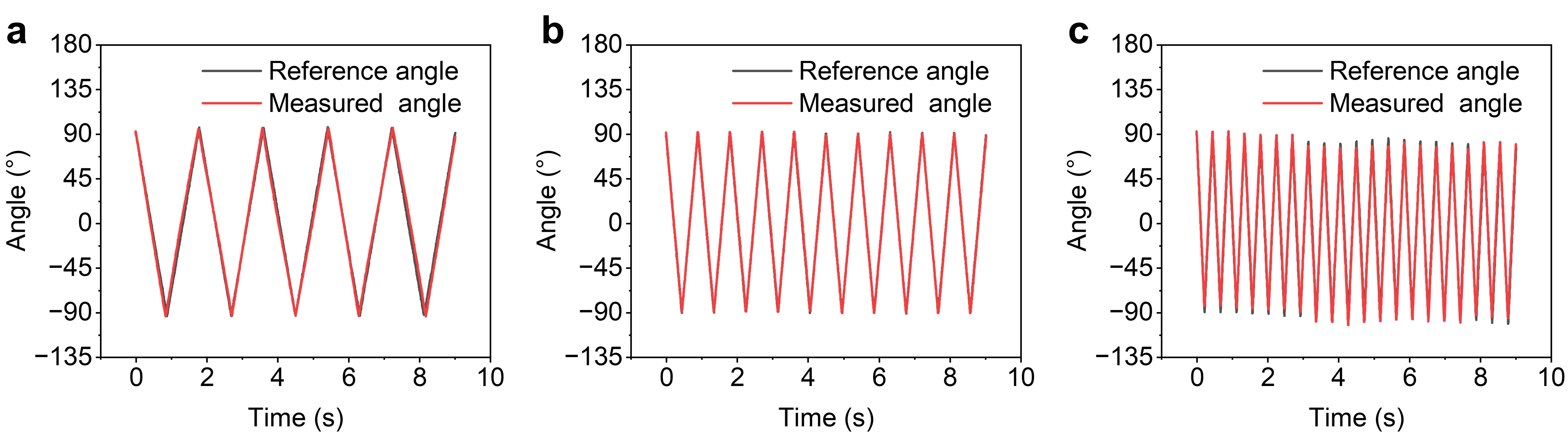


**Figure S17.** Comparison of the measured angle between the IMUs and the actual angle of the servo output under triangular wave conditions with speeds of a) 200°/s, b) 400°/s, and c) 800°/s.

**Figure S18.** Temperature profiles of the smart adhesive pad and the inner surface of the glove during the heating and cooling process.

**Supplementary Videos**

**Movie S1.** Example of performance of the drinking task performed by the subject: Unassisted vs Assisted.mp4

**Movie S2.** Demonstration of grasping ability improvement in activities of daily living of the subject: Unassisted vs Assisted.mp4
